# Targeted expression of step-function opsins in transgenic rats for optogenetic studies

**DOI:** 10.1038/s41598-018-23810-8

**Published:** 2018-04-03

**Authors:** Hiroyuki Igarashi, Keiko Ikeda, Hiroshi Onimaru, Ryosuke Kaneko, Kyo Koizumi, Kaoru Beppu, Kayo Nishizawa, Yukari Takahashi, Fusao Kato, Ko Matsui, Kazuto Kobayashi, Yuchio Yanagawa, Shin-Ichi Muramatsu, Toru Ishizuka, Hiromu Yawo

**Affiliations:** 1 Department of Physiology and Pharmacology, Tohoku University Graduate school of Medicine, Sendai, Japan; 2 Department of Physiology, International University of Health and Welfare, Narita, Chiba, Japan; 3 Division of Biology, Center for Molecular Medicine, Jichi Medical University, Shimotsuke, Tochigi, Japan; 4 Department of Physiology, Showa University School of Medicine, Shinagawa, Tokyo, Japan; 5 Department of Genetic and Behavioural Neuroscience, Gunma University Graduate School of Medicine, Maebashi, Japan; 6 Department of Developmental Biology and Neuroscience, Tohoku University Graduate School of Life Sciences, Sendai, Japan; 7 Department of Super-network Brain Physiology, Tohoku University Graduate School of Life Sciences, Sendai, Japan; 8 Department of Molecular Genetics, Institute of Biomedical Sciences, Fukushima Medical University School of Medicine, Fukushima, Japan; 9 Department of Neuroscience, The Jikei University School of Medicine, Tokyo, Japan; 10 Division of Neurology, Jichi Medical School, Tochigi, Japan; 11 Center for Gene & Cell Therapy, The Institute of Medical Science, University of Tokyo, Tokyo, Japan; 12 Present Address: Research Fellow of the Japan Society for the Promotion of Science (JSPS Research Fellow), Tokyo, Japan

## Abstract

Rats are excellent animal models for experimental neuroscience. However, the application of optogenetics in rats has been hindered because of the limited number of established transgenic rat strains. To accomplish cell-type specific targeting of an optimized optogenetic molecular tool, we generated ROSA26/CAG-floxed STOP-ChRFR(C167A)-Venus BAC rats that conditionally express the step-function mutant channelrhodopsin ChRFR(C167A) under the control of extrinsic Cre recombinase. In primary cultured cortical neurons derived from this reporter rat, only Cre-positive cells expressing ChRFR(C167A) became bi-stable, that is, their excitability was enhanced by blue light and returned to the baseline by yellow~red light. In bigenic pups carrying the Phox2B-Cre driver, ChRFR(C167A) was specifically expressed in the rostral parafacial respiratory group (pFRG) in the medulla, where endogenous Phox2b immunoreactivity was detected. These neurons were sensitive to blue light with an increase in the firing frequency. Thus, this transgenic rat actuator/reporter system should facilitate optogenetic studies involving the effective *in vivo* manipulation of the activities of specific cell fractions using light of minimal intensity.

## Introduction

Understanding the neural system of the brain by revealing the interconnected networks and their emerging functions is a major aim of neuroscience. Previously, the mapping of brain circuits was conducted using pharmacological or electrical stimulation methods. Although electrical stimulation enables temporally precise control of the neuronal activity, the spatial resolution is not high because of the widespread electric field in brain tissue. In addition, it is difficult to differentially stimulate a specific type of neuron in the network. By contrast, the discovery of channelrhodopsins, algal photoreceptor proteins^1,2^, prompted the generation of photosensitive neurons genetically engineered to express channelrhodopsins^3,4^. This state-of-the-art technology, referred to as optogenetics, is now indispensable to modern neuroscience owing to its genetic selectivity, high spatiotemporal precision and low invasiveness^5–8^. When extending this method to animal models, both the satisfactory expression of engineered opsin genes in a genetically defined cell type and the delivery of light at a specific wavelength must be achieved^9,10^.

Recently, various transgenic mice have been created to enable optogenetic studies of the neural mechanisms underlying behaviours^11,12^. However, larger rodent models are required to investigate higher-order brain functions. The rat is a favourable non-human model system^13^ that offers potential advantages over mice because of their larger body size and superior ability to accomplish complex behavioural paradigms^14,15^. For example, rats can accommodate a large number of electrodes and LED fibres to record the resultant neuronal activity from multiple sites *in vivo* in response to the stimulation of multiple neurons^16^.

One of the original representative transgenic rat strains expressing an optogenetic molecular tool is the thy1.2-ChR2-Venus line (W-TChR2V4)^17–20^. Although this line can enable the specific targeting of ChR2 to retinal ganglion cells, dorsal root ganglion neurons, and various neurons in the hippocampus and cerebral cortex under the regulation of the thy1.2 promoter, a conditional expression system with optimized opsins is needed to establish a generalizable approach. With respect to the conditional expression system, a panel of the Cre recombinase-driver rat lines was introduced for the optogenetic targeting of genetically defined cell types using virus vectors incorporating the genes of interest in the downstream portion of relatively strong promoters^8,21^. However, since this method relies on a virus injection strategy, the number and location of neurons that express the optogentic molecular tools often diverge among animals. Therefore, practical reporter strains of rats that conditionally express optogenetic molecular tools under a Cre-loxP system are highly desired.

Regarding the optimization of opsin genes, many natural opsins have been identified, and their utilization has been improved through molecular modifications^6,10,22^. Among these molecules, point mutations of the so-called DC gate, comprising C128 and D156 of ChR2 or their counterparts, substantially affect the photocycle kinetics of channelrhodopsins to become bi-stable (step-function opsins, SFOs). That is, the step-function mutants are typically activated in response to light, such as blue light, remaining open in the dark for an extended period and shutting down in response to another light, such as yellow or violet light^23,24^. Thus, step-function mutants are highly sensitive to dim light and behave like photon accumulators, making these molecules attractive candidates for *in vivo* optogenetics using large animals.

Here, we generated a novel transgenic actuator/reporter rat strain expressing one of the well-characterized step-function mutants of chimaera channelrhodopsins, ChRFR(C167A)^25^, in the presence of exogenous Cre recombinase (ROSA26/CAG-floxed STOP-ChRFR(C167A)-Venus BAC rat). The ChRFR(C167A)-expressing neurons from this rat were sensitive to dim blue light, increasing neural excitabilities both *in vitro* and in the brain *ex vivo*. These new transgenic rat strains will contribute to a wide range of biomedical studies through the optogenetic modulation of targeted cellular activity *in vivo*.

## Results

### Cre-dependent expression of ChRFR(C167A)-Venus

**Figure 1:**
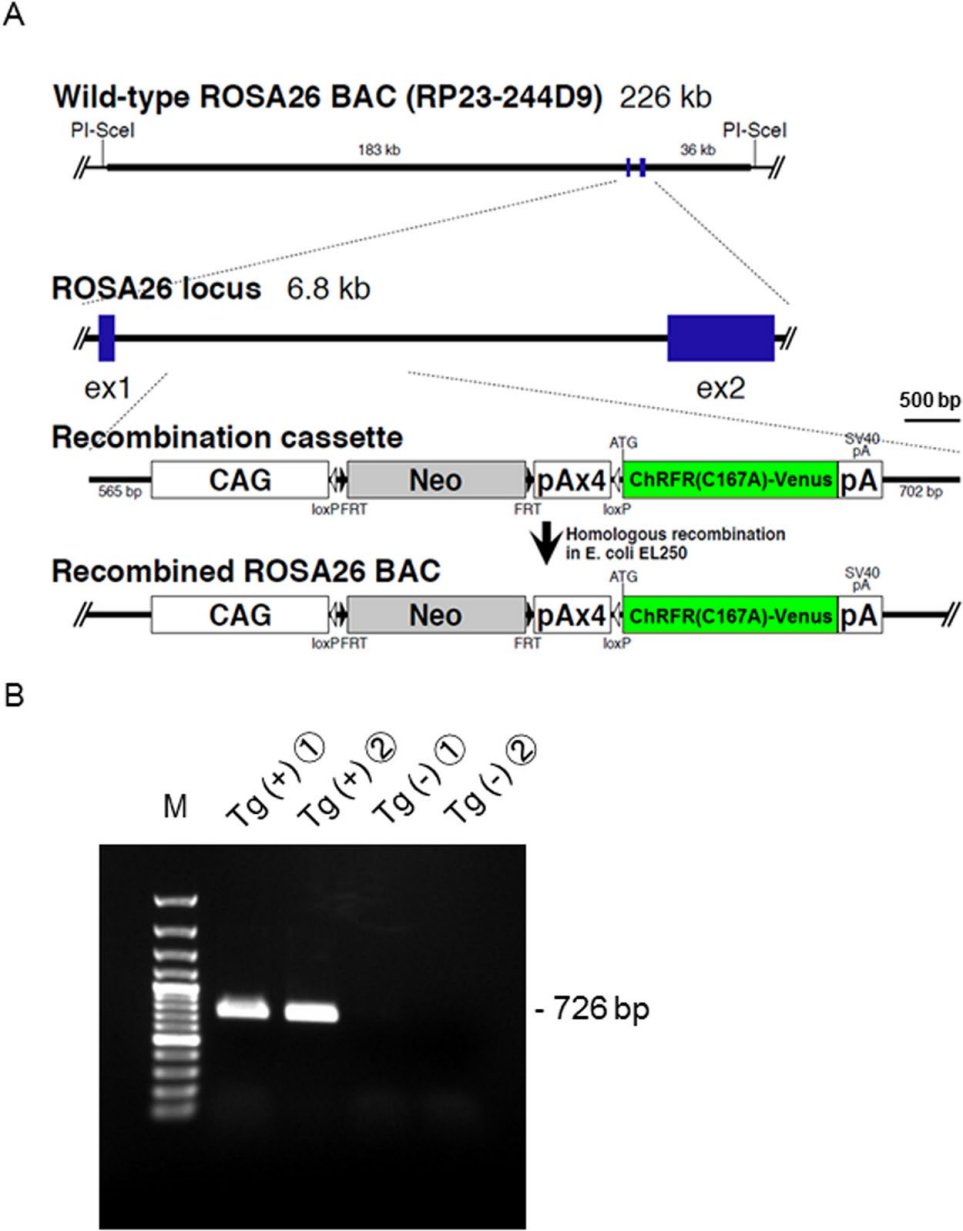
Generation of ROSA26/CAG-floxed STOP-ChRFR(C167A)-Venus BAC actuator/reporter rats. (**A)** The transgene construct, which was inserted into the mouse ROSA26 BAC clone, comprised the CAG promoter, a cassette for the neomycin resistance gene with four STOP signal repeats (pA) flanked by loxP sites, and a sequence containing the ChRFR-C167A open reading frame. After Cre-mediated recombination, ChRFR(C167A)-Venus expression was under the regulation of the generic CAG promoter. (**B**) PCR analysis of genomic DNA. The 726-bp band, which is recognized by the designed primer sets targeting the PGK-neo cassette sequence between two loxP sequences, was present in the ChRFR(C167A)-Venus reporter rats but not in the wild-type rats. A 100-bp DNA ladder was used as marker (M). A full uncropped image is available as Supplementary Figure S1A.

We established and characterized a transgenic actuator/reporter rat strain, (ROSA26/CAG-floxed STOP-ChRFR(C167A)-Venus BAC rat), carrying three copies of genome constructs with the ChRFR(C167A)-Venus gene downstream of a floxed STOP cassette containing the neomycin–resistant gene and four repeats of the polyadenylation sequence (Fig. 1A). The gene was controlled under the cytomegalovirus enhancer/chicken β-actin promoter (CAG promoter) at the ROSA26 site of a mouse BAC containing the ROSA26 gene^26^. After loxP site-specific excision of the STOP cassette, ChRFR(C167A) tagged with the yellow fluorescent protein Venus^27^ was expressed under the CAG promoter. PCR analysis confirmed that the transgenic (Tg) rats possessed a neo cassette. Rats with the 726-bp band were considered floxed STOP-positive and used for further experiments (Fig. 1B).

### Actuator/Reporter gene expression by the transfection of exogenous Cre gene

**Figure 2:**
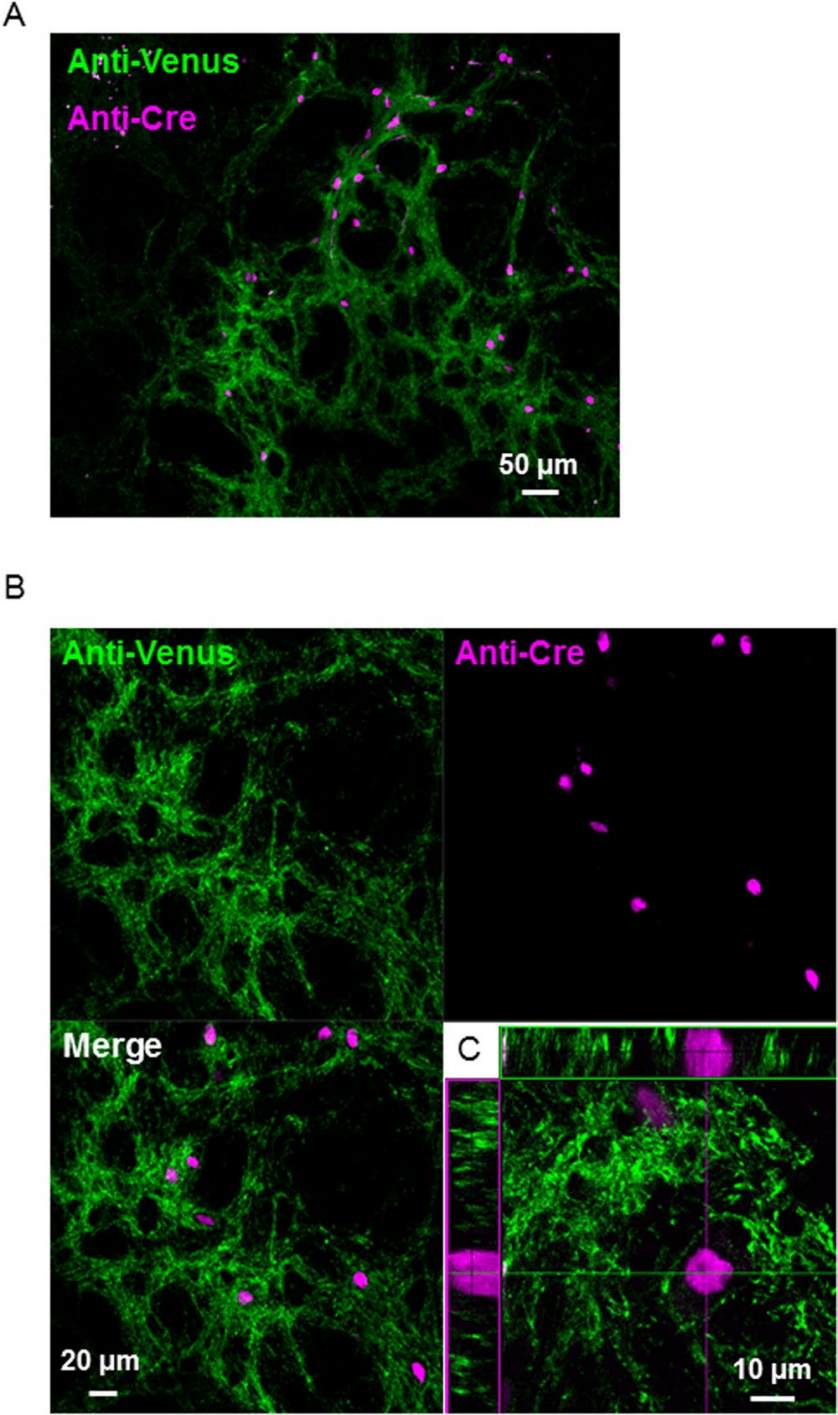
Viral vector-mediated reporter gene expression in striatum. (**A**) Confocal image of brain sections stained with antibodies against Cre (magenta) and GFP (Green). The ROSA26/CAG-floxed STOP-ChRFR(C167A)-Venus BAC actuator/reporter rat was injected with AAV2-Cre in its striatum. (**B**) Localization of Cre-immunoreactive nuclei (magenta) and Venus immunoreactivity (green) in neurons at striatum. (**C**) Orthogonal view on the brain image stack of a neuron which has Cre-positive nucleus. Note that Venus accumulated in the plasma membrane and did not merge with the Cre signals.

To examine the conditional expression of ChRFR(C167A)-Venus, we injected adeno-associated virus 2 (AAV2)-*Cre* virus vectors into the striatum or hippocampus of heterozygous rats (*n* = 3) obtained by crossing male ROSA26/CAG-floxed STOP-ChRFR(C167A)-Venus BAC rats with wild-type female Long-Evans (LE) rats. At 3 weeks post-injection, Venus fluorescence was detected at all injection sites, and the signals were closely confined to the injection points (Fig. 2A, Supplementary Fig. S2). By contrast, the expression of Venus was not detected on the other side of the hemisphere, which underwent PBS injection. At higher magnification, Cre signals were clearly detectable in the nuclei, and Venus signals were accumulated in the plasma membrane of those cells; importantly, these signals did not merge with each other (Fig. 2B).

### Robustness of the actuator/reporter system

**Figure 3:**
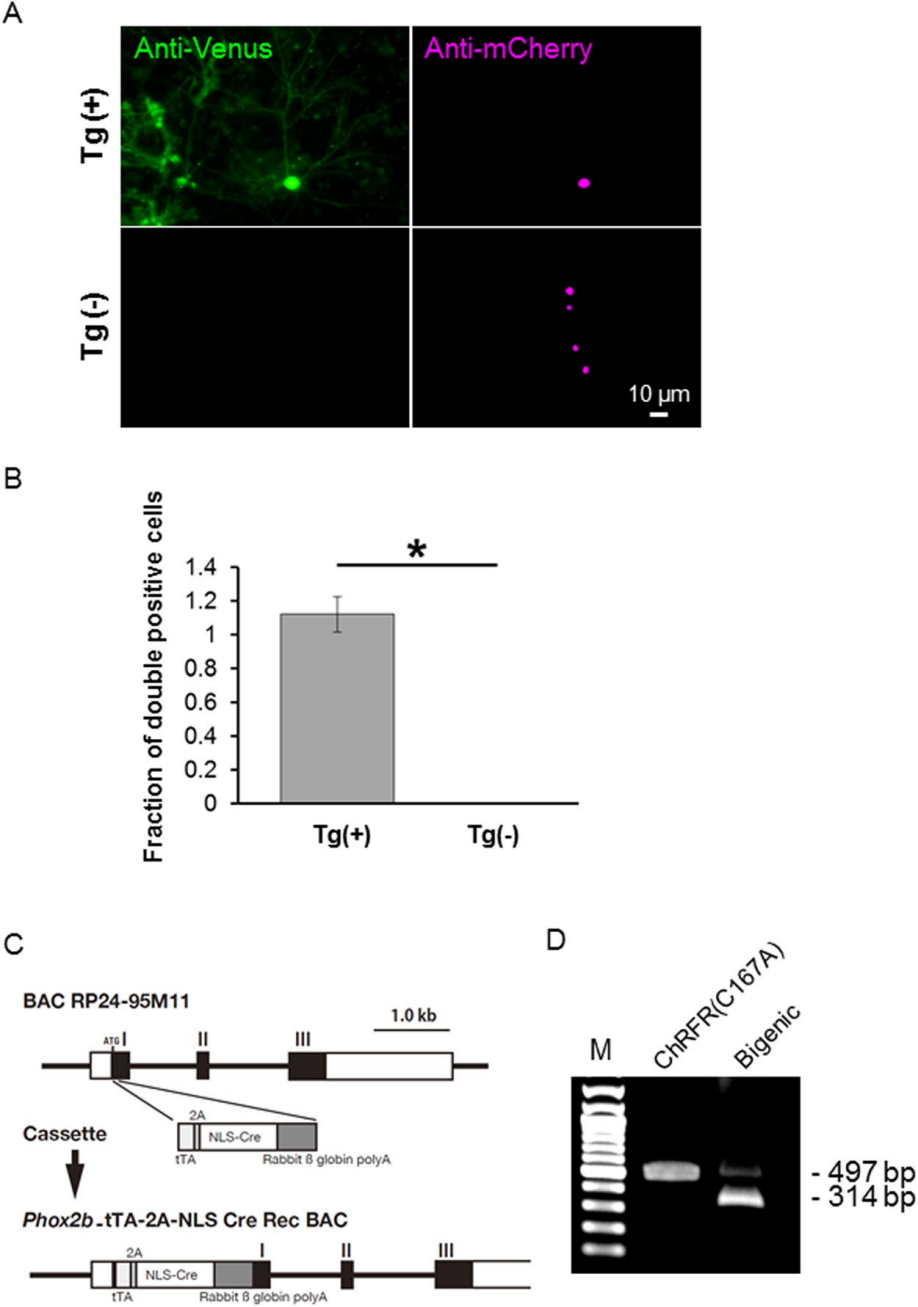
Robustness of the reporter system. (**A**) The primary cultured cortical neurons were prepared from both Tg-positive and negative littermates, and mCherry-NCre was transfected using calcium phosphate method; Venus fluorescence (green) and mCherry-NCre (magenta). (**B**) Comparison of the fraction of double-positive cells with ChRFR(C167A)-Venus and mCherry-NCre between littermates (Tg, *n* = 5 pups; wild-type, *n* = 6 pups). For each group, 25 fields of interest were randomly chosen, and the cell number was counted in a double-blind manner. *p < 0.001, Student’s paired *t*-test. (**C**) Structure of the *Phox2b*_tTA-2A-Cre BAC transgene. The exon-intron structure of the *Phox2b* gene is shown in the upper part. The first exon that contains noncoding (a white rectangle) and coding (a black rectangle) regions, the second exon (a black rectangle), and the third exon which contains coding (a black rectangle) and noncoding (a white rectangle) regions are indicated as I, II, and III, respectively. The recombination targeted the first exon (around the ATG site of *Phox2b* gene) by inserting the tTA, 2 A peptide, and Cre recombinase with nuclear localization signal (NLS) coding sequences attached the rabbit ß globin gene polyadenylation sequence at 3′ end. (**D**) PCR assay of the genomic DNA extracted from the homogenized whole brain of a ChRFR(C167A)-Venus actuator/reporter rat. The allele of BAC with STOP sequence was detectable as 497 bp band. In the bigenic rat with Phox2B-Cre driver, 314 bp band became manifest as expected by the removal of Neo-STOP sequence. A full uncropped image is available as Supplementary Figure S1B.

To validate the Cre-dependent expression in this actuator/reporter line, primary cultured cortical neurons of ROSA26/CAG-floxed STOP-ChRFR(C167A)-Venus BAC rats were transfected with plasmids containing *mCherry-NCre*^28^. The mCherry-positive nuclei and Venus-positive plasma membranes were detected only in cells (n = 28) prepared from Tg-positive embryos (n = 5). By contrast, cells (n = 59) expressing mCherry-NCre prepared from Tg-negative siblings (n = 6) did not express ChRFR(C167A)-Venus (Fig. 3A,B). An unexpected STOP excision in loxP-based reporter animals is referred to as leaky expression^29,30^. To detect leaky expression in this actuator/reporter system, the DNA template was extracted from homogenized whole brains of ROSA26/CAG-floxed STOP-ChRFR(C167A)-Venus BAC rats, and each allele in the BAC construct, with (497 bp) or without the STOP sequence (314 bp), was amplified using PCR. As a positive control, the DNA template extracted from the medullary tissue of bigenic pups obtained by crossing ROSA26/CAG-floxed STOP-ChRFR(C167A)-Venus BAC rats and *Phox2b*_tTA-2A-*Cre* driver rats (Fig. 3C) was used. Only a single band corresponding to the allele with the STOP sequence was detected in the actuator/reporter rat, indicating that background recombination was negligible (Fig. 3D). In subsequent experiments, a subpopulation of cortical neurons expressing ChRFR(C167A)-Venus under the control of the CaMKIIα promoter was used for the whole-cell patch clamp experiments.

### Regulation of neuronal activity using two colours of light

**Figure 4:**
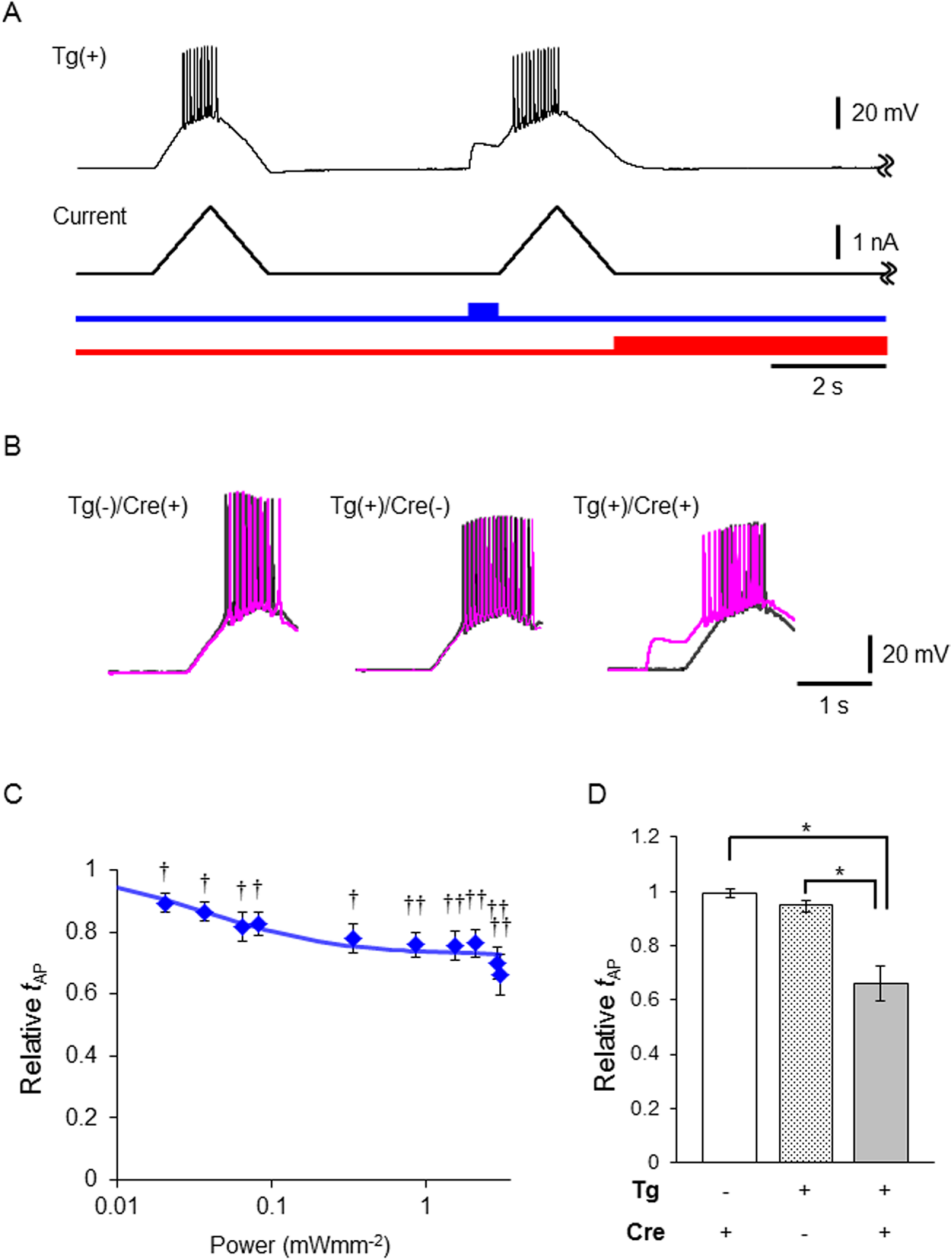
Optical manipulation of excitability of neurons from the ROSA26/CAG-floxed STOP-ChRFR(C167A)-Venus BAC actuator/reporter rat. (**A**) Typical recordings from the primary cultured cortical neurons in which ChRFR(C167A)-Venus was expressed by the transfection of CaMKIIα-mCherry-NCre plasmid: (top) the recording from transgenic positive (Tg(+)) neuron, (middle) current ramp injection, (bottom) Light stimulation paradigm for blue light (blue line, 438 ± 24 nm, 3 mWmm^−2^) and red light (red line, 643 ± 20 nm, 6.8 mWmm^−2^). (**B**) Overlay of representative traces of current injection without (black) and with blue light irradiation (magenta). (**C**) Relative time to evoke the first action potential (*t*_AP_) to that without light was calculated for each trace and plotted as a function of light power density. The change of this value was statistically tested using paired raw data before calculation using Wilcoxon signed rank test (^†^P < 0.01 and ^††^P < 0.001). The fitting line was drawn according to the Michaelis-Menten relationship (*K*_*d*_ = 0.04 mWmm^−2^, *V*_*max*_ = 0.27). (**D**) Summary of relative *t*_AP_ value (*t*_AP_ of light(+)/*t*_AP_ of light(−)) in each population: Tg(−)/Cre(+),Tg(+)/Cre(−) and Tg(+)/Cre(+). *P < 0.0005, Mann-Whitney *U*-test. Values are mean ± SEM.

It was well anticipated that the excitability of a neuron from the ROSA26/CAG-floxed STOP-ChRFR(C167A)-Venus BAC rats could be manipulated by light when it expressed Cre. The excitability of a neuron in the primary culture was tested by current ramp injections under whole-cell current clamp (Fig. 4A). At first, the slope of a ramp was adjusted to generate several action potentials. Next, the same current ramp was injected after illumination with blue light (500 ms, 438 ± 24 nm) and then red light (8 s, 643 ± 20 nm, 6.8 mWmm^−2^) starting after the current ramp injection. In every Cre-positive neuron (n = 16), the membrane potential was depolarized solely by blue light in a manner dependent on the power (Supplementary Fig. S3). Although this depolarization was accompanied by action potentials in two of them, it was not in the others (n = 14) even at the maximal power of light (3 mWmm^−2^). However, the time to evoke the first action potential (*t*_AP_) was significantly reduced during the current ramp injection by the preceding blue light in the Cre-positive neurons, but not in the Cre-negative neurons (Fig. 4B,C). On the other hand, when the primary culture was made from the littermate embryo without the transgenic allele, neither the depolarization nor the reduction of *t*_AP_ was induced by any light even in the Cre-positive neurons (Fig. 4D).

### Directive expression using Phox2B Cre-driver rats

**Figure 5:**
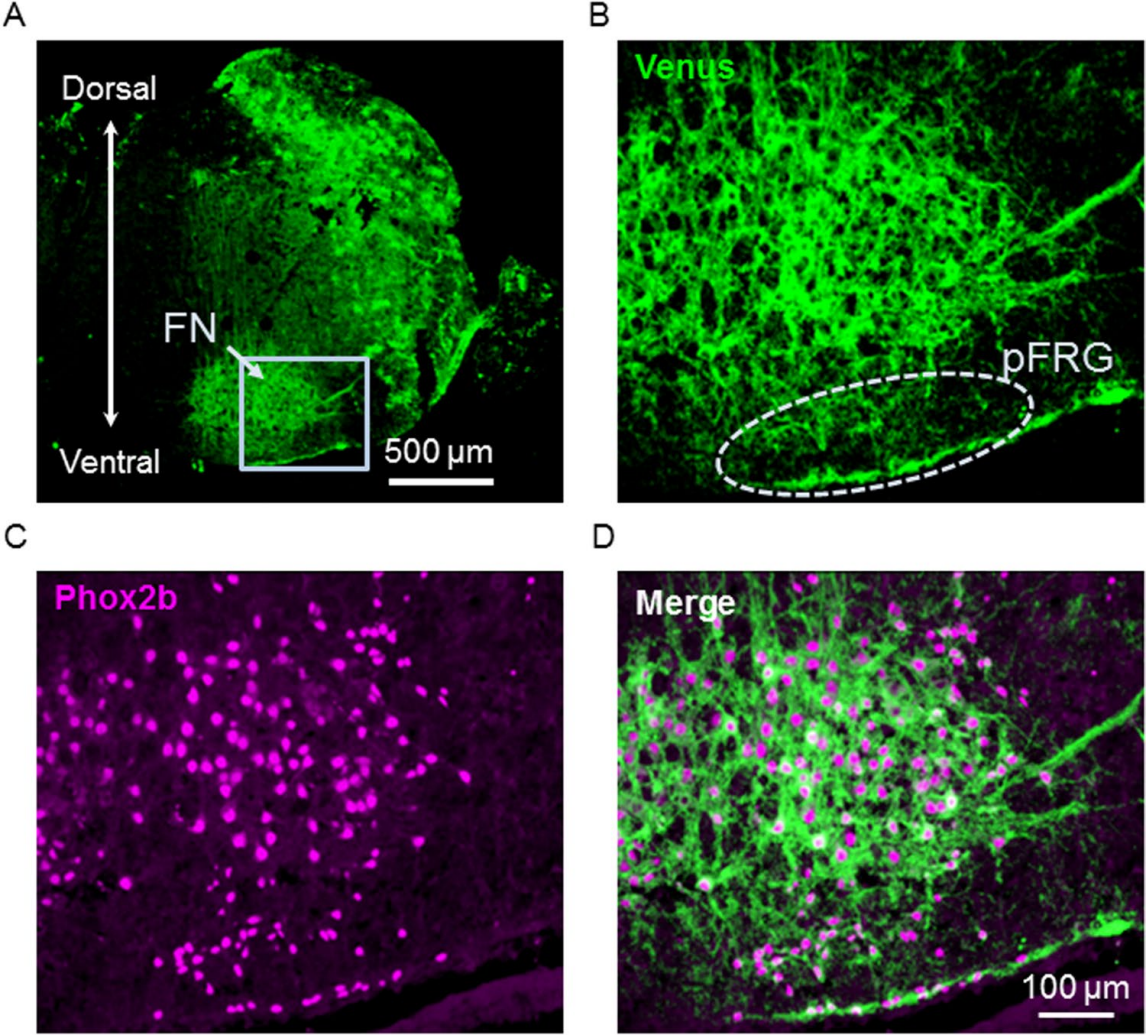
Expression of ChRFR(C167A)-Venus in the bigenic rat with Phox2B-Cre driver. (**A**) Venus fluorescence image at lower magnification. FN, facial nucleus; pFRG, parafacial respiratory group. The light blue square denotes the area of B-D. (**B)** Venus; (**C)** anti-Phox2b immunofluorescence; (**D)** merge. Cell group of the pFRG is in an area indicated by a dotted line in B. Most Phox2b positive cells express Venus.

**Figure 6:**
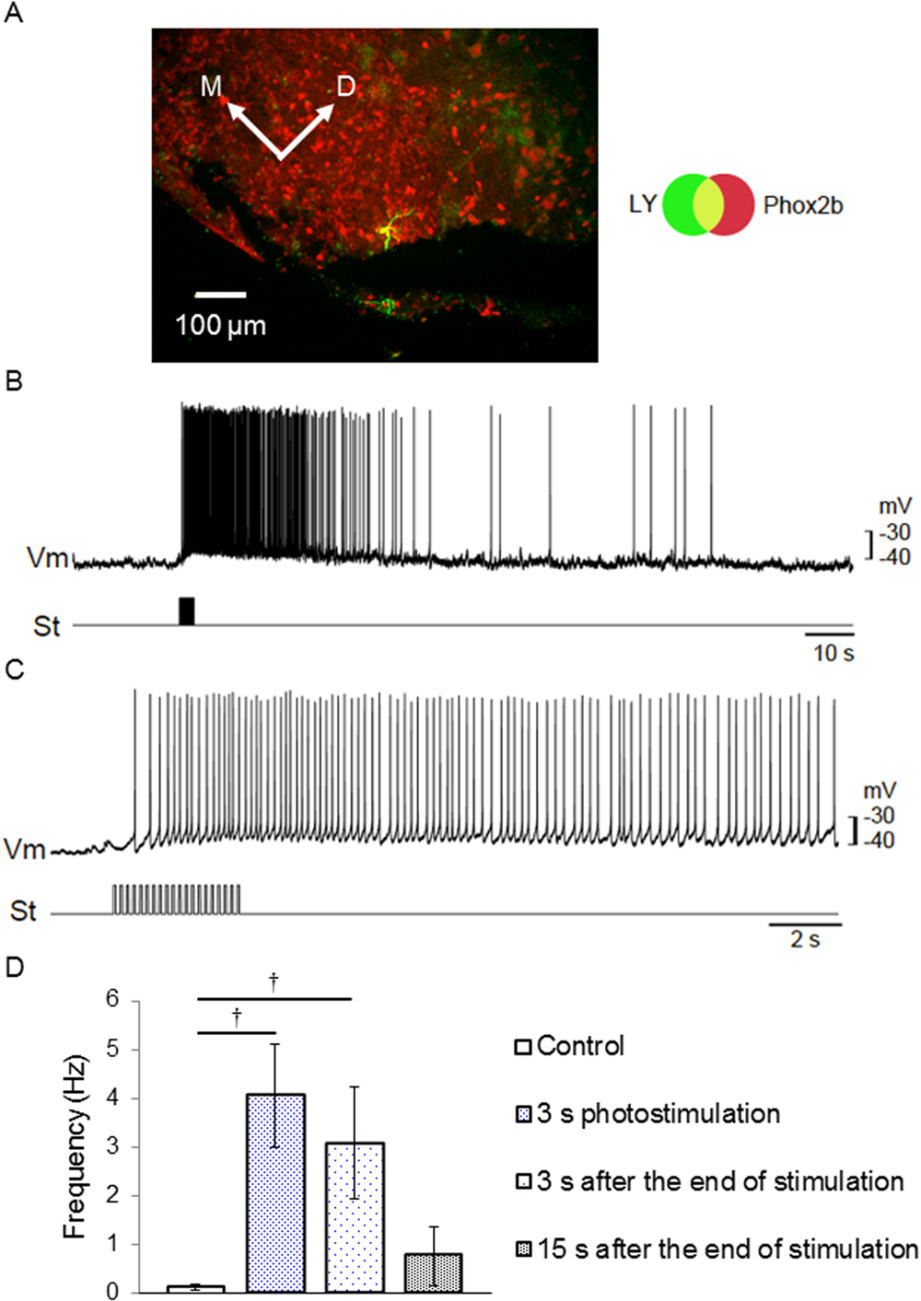
Optical manipulation of neural excitability in the central nervous sytem (CNS). (**A)** A sample pFRG neuron which was immunoreactive with Phox2b (red) and stained with Lucifer yellow in the patch electrode (green). (**B**) membrane potential trajectory during photostimulation (460–470 nm, 0.1–0.2 mWmm^−2^, duration 50 ms, interval 150 ms, 6.7 Hz). Upper trace (Vm), membrane potential; lower trace (St), photostimulation given as a train of light pulses. (**C**) Similar to B, but the response of another neuron to shorter train of photostimulation pulses. Note that the time scale is different. (**D**) Summary of neuronal excitability (n = 6). The firing frequency was compared at different time points of photostimulation: before photostimulation (the first column), 3 s after photostimulation (the second column), 3 s after end of photostimulation (the third column) and 15 s after end of photostimulation. ^†^P < 0.005, Wilcoxon signed rank test.

*Phox2b* is a paired-like homeobox-encoding gene and a key regulator of autonomic neural crest derivatives and placode-derived visceral sensory ganglia. *Phox2b* is also expressed in pre-inspiratory (Pre-I) neurons in the parafacial respiratory group, constituting one of the respiratory rhythm generators in the medulla of newborn rats^31,32^. We previously reported that the expression of PHOX2B is a remarkable feature of Pre-I neurons and a useful marker for identifying the cytoarchitecture of neurons in the pFRG in the newborn rat medulla^33^. Newborn pups expressing ChRFR(C167A)-Venus under the control of Phox2b enhancer/promoter were obtained by crossing ROSA26/CAG-floxed STOP-ChRFR(C167A)-Venus BAC rats with *Phox2b*_tTA-2A-*Cre*-driver rats (Fig. 3C). The functional expression of ChRFR(C167A) was evaluated in the newborn, bigenic rat for the following three issues. First, we investigated whether the Venus-positive signals driven by Cre recombinase could be observed in Pre-I neurons of the pFRG. A Venus-positive signal was detected in the rostral pFRG in the superficial area just ventral to the facial nucleus (FN) in the medulla, where endogenous Phox2b immunoreactivity was detected (Fig. 5). Second, we compared the expression pattern of ChRFR(C167A)-Venus in Phox2b-Cre/floxed ChRFR(C167A) with that of tdTomato in Phox2b-Cre/floxed STOP tdTomato bigenic neonatal rats^15^ (Supplementary Figs S4, S5). The expression of ChRFR(C167A)-Venus was consistently detected, and the expression patterns of the actuator were coincident with those of tdTomato throughout the brainstem-spinal cord sections. The reliability of this Cre-dependent recombination was further evaluated using VGAT-*Cre* BAC rat (NBRP-0839, Supplementary Fig. S6). Even in other brain regions such as the hippocampus, cortex, cerebellum, and striatum in the bigenic rats, the expression of ChRFR(C167A)-Venus was successfully induced and coincident with the distribution of GABAergic neurons. Third, the effects of photostimulation were examined on the Phox2b positive pFRG neurons in the *ex vivo* brainstem-spinal cord preparation (n = 6, Fig. 6). The Phox2b-positive neurons were depolarized in the membrane potential with increased excitability during and after photostimulation by blue LED. Indeed, the firing frequency was significantly increased upon blue light stimulation. The neuronal excitability was up-regulated for several seconds after the end of photostimulation, but returned to baseline in tens of seconds (Fig. 6D).

## Discussion

Here, we demonstrated the efficient and sensitive Cre-mediated expression of actuator/reporter genes in ROSA26/CAG-floxed STOP-ChRFR(C167A)-Venus BAC rats. The strict regulation of ChRFR(C167A)-Venus expression by Cre recombinase was supported on the following grounds: (1) ChRFR(C167A) conjugated with the fluorescent marker Venus was expressed only in neurons transfected with *Cre* after AAV2-*Cre* injection; (2) ChRFR(C167A)-Venus was exclusively expressed in primary cultured cortical neurons containing *Cre*; and (3) ChRFR(C167A)-Venus was only expressed in neurons expressing Cre under a cell type–specific promoter using the *Phox2b*_tTA-2A-NLS-*Cre* BAC and VGAT-*Cre* BAC transgenic strain. Our results indicate that the cells carrying the active promoter could be selectively labelled with Venus fluorescence and effectively depolarized by light by combining these actuator/reporter rats with an appropriate driver rat strain. Although ChRFR(C167A) was well characterized in the photocurrent kinetics and its photocurrent was more dependent on Na^+^ and Ca^2+^ than H^+^ like ChR1 and 2^34,35^ (Supplementary Fig. S8A and S8B, Supplementary Table S2), the validity of this system should be checked in each case for the practical expression in various subpopulations of neurons. It should also be noted that the ON/OFF kinetics of a photocurrent is dependent on the cellular environment such as temperature and pH^2^ as well as the molecular state of rhodopsin^36,37^.

Rats and mice have frequently been used as fundamental model organisms for biomedical research. Although similar in many aspects, there are several obvious differences between these rodent species in functional anatomy, physiology, and pharmacology^38^. Interestingly, the characteristics of Phox2b-expressing cells in the parafacial region of neonatal rats are basically similar to those in mouse, but the roles in respiratory rhythm generation during development are distinct^39^. Thus, the establishment of a reliable transgenic system in rats for further studies has been desired, and the two BAC Tg lines introduced herein, namely, ROSA26/CAG-floxed STOP-ChRFR(C167A)-Venus BAC rats and *Phox2b*_tTA-2A-*Cre* driver rats, would contribute to expanding the repertoire and offer an attractive means for precise optogenetic targeting.

The application of an optogenetic approach to larger animal models has been challenging because the light has to be widely delivered to activate an entire substructure of larger size. To overcome this problem, the animal model has to accommodate a substantial number of modified optic fibres that deliver strong light into a wider region in the tissue, or the photocurrent properties, including the light sensitivity of the opsins, have to be optimized. In one of the well-characterized step-function variants of ChRs, ChRFR(C167A), the magnitude of the photocurrent was approximated by a function of the turning-on (ON) rate constant, which is equivalent to τ_ON_^−1^, and the irradiation time^25^. Therefore, even light at a weak power could effectively depolarize the neuronal membrane to become excitable with prolongation of the irradiation time. In the present study, ChRFR(C167A) was highly sensitive to blue light (50% activation; 0.08 mWmm^−2^, Supplementary Fig. S3), which was less than 1/10 of the power required for 50% activation of native channelrhodopsin-2 (1.3 mWmm^−2^)^40^. When all synaptic inputs of the cultured cortical neurons from our ROSA26/CAG-floxed STOP-ChRFR(C167A)-Venus BAC rats were pharmacologically blocked, the light-evoked depolarization was often subthreshold even with the maximal power in the Cre-positive neurons. However, the excitability of a neuron was actually increased by light as evidenced by the reduction of *t*_AP_. That is, the light should cause some of the sub-threshold EPSPs to become excitable in neurons in the network. Indeed, the irradiation of weak blue light (0.1-0.2 mWmm^−2^) onto the brainstem-spinal cord of rat neonates was sufficient to enhance the excitability of neurons in the pFRG (Fig. 6). To our knowledge, this actuator/reporter system is the first successful use of a step-function mutant channelrhodopsin in a transgenic rat. With the temporally precise tuning of the membrane potentials, this rat line is optimized for an experimental context that requires modulation of the spontaneous firing rate rather than the generation of every action potential^23,41^. For instance, the transition from a hyperpolarized to a depolarized state of the membrane potential is usually accompanied by a peculiar pattern of electrical activity intrinsic to its neuronal subclass^42,43^. The relevance of the periodic oscillations in a genetically defined cell population to the specific behavioural outcome could be directly tested by simply alternating the delivery of blue or red light into the targeted region^44^.

In summary, this actuator/reporter rat strain enables the control of identified neuronal activity by light under the visualization of Venus fluorescence. It appears to be suitable to optically manipulate deep in the tissue, since the spectral sensitivity of ChRFR(C167A) is more red-shifted than the SFOs derived from ChR2^25^ and the brain tissue preferentially absorbs short wavelengths of light^45^. With the above-mentioned advantages and strengths of the relatively large body size and complex behaviour of rats, the transgenic lines reported herein would facilitate a variety of medical studies, including neuroscience.

## Methods

### Construction of ROSA26/CAG-floxedSTOP-ChRFR(C167A)-Venus BAC

The ROSA26/CAG-floxed STOP-ChRFR(C167A)-Venus BAC was generated using a BAC recombineering method^15,46^ with a BAC clone containing the mouse ROSA26 locus. Briefly, a BAC clone (RP23-244D9) from a C57BL/6 J mouse genomic BAC library (BACPAC Resource Center, Oakland, CA, USA) spanning ~183 kb upstream of ROSA26 exon 1 to ~34 kb downstream of ROSA26 exon 3 (total size: ~226 kb) was used. To modify the Rosa26 locus, a cassette containing the following components was constructed: a CAG promoter derived from pCAGGS^47^, loxP, FRT-flanked Kan/Neo cassette (from J. Takeda, Osaka University), 4 × poly(A) (from M. Yamamoto, Gunma University), ChRFR-C167A cDNA^48^, and SV40 poly(A). Through lambda red protein-mediated homologous recombination in *E*. *coli* EL250^49^, the above-mentioned cassette flanked by homologous fragments was inserted into the ROSA26 BAC clone at the desired location. The following primers were used to generate 5′ and 3′ homology regions: R5-FS, gtcgaCGTCGTCTGATTGGCTCTC and R5-RX, ctcgaGACTGGAGTTGCAGATCAC for the 5′ homology region; and R3-FS, gtcgACAGTGTCGCGAGTTAGA and R3-RX, ctcgagCACCTGAACTTTGCATTCC for the 3′ homology region. The recombinants were identified after screening for kanamycin resistance, followed by PCR analysis. The following primers were used to assess the integrity of the recombination: R5upF2, CGTCTCGTCGCTGATTGGCTTC and CAG-R2, CCGTAAATAGTCCACCCATTGACG for the 5′ site; and G/RFPtail-F, CATGGACGAGCTGTACAAG and R3dwnR2, ATGCCATGAGTCAAGCCAG for the 3′ site. The loxP site and lox511 site in the pBACe3.6 backbone were removed, and the PI-SceI site was inserted using a BAC recombineering method with the PIsac-mLK cassette (from R. Kaneko, Gunma University) or the d4PI511zeo cassette (from R. Kaneko, Gunma University), respectively.

### Generation of ROSA26/CAG-floxedSTOP-ChRFR(C167A)-Venus BAC Tg rats

Recombined ROSA26/CAG-floxed STOP-ChRFR(C167A)-Venus BACs were linearized by PI-SceI digestion and subsequently gel purified^50^. The purified BAC fragment was microinjected into the pronuclei of Long–Evans (LE) rat oocytes as previously described^49^. Among the 289 oocytes injected, 238 cells could be transferred into pseudo-pregnant female rats, and 52 pups were obtained. Nine ROSA26/CAG-floxed STOP-ChRFR(C167A)-Venus BAC Tg founders were identified from the 52 weaned pups, and one of the lines is described herein. The BAC transgenic rats were maintained on an LE genetic background (Institute for Animal Reproduction, Ibaraki, Japan) and deposited to the National BioResource Project for the Rat in Japan (NBRP-Rat) (http://www.anim.med.kyoto-u.ac.jp/nbr/default.aspx) as LE-Tg(Gt(ROSA)26Sor-CAG-COP4*C167A/YFP*)1Jfhy (NBRP-0840).

### Detection of transgene recombination

Genomic DNA was extracted using a standard method^51^ or simple alkali isolation. In the alkali extraction, the animal tissue was immersed in 50 mM NaOH and subsequently vortexed. After heating for 10 min at 95 °C, the solution was neutralized using 1 M Tris-HCl, followed by centrifugation. Only the supernatant was used for the subsequent PCR experiments. Genomic DNA was used as the template for PCR to detect the transgene using the following primer: 5′-CTATGACTGGGCACAACAGACAAT-3′ (in the Neo resistance gene-coding region). To evaluate the effectiveness of recombination, DNA was extracted from homogenized whole brains. Each allele with/without a STOP sequence was PCR-amplified using either set of primers: pCX1624F, CTAGAGCCTCTGCTAACC and PGK-R, GACGTGCTACTTCCATTTGTCAC for the STOP-remaining allele (PCR product: 497 bp); or pCX1624F, CTAGAGCCTCTGCTAACC and ChR + 136 R, CTCGGTGGAAGACGTAATCAGG for the STOP-deleted allele (PCR product: 314 bp). The PCR fragments were amplified at 95 °C for 3 min, followed by 35 cycles at 95 °C for 30 sec, 60 °C for 30 sec, and 72 °C for 1 min using KOD FX Neo (TOYOBO, Osaka, Japan). GeneRuler 100 bp DNA ladder (Fermentas, Burlington, ON, Canada) was used as marker for electrophoresis.

### Viral expression of Cre recombinase

For histological evaluation, the rats were anaesthetized using sodium pentobarbital (50 mg/kg, i.p.) and were injected with the viral vector. The vector solution (0.5 μl, 1.03 × 10^13^ vector genomes/ml) or PBS (0.5 μl/site) was injected into 8 sites of the dorsal striatum through a glass microinjection capillary connected to a micro-infusion pump (ESP-32; Eicom, Kyoto, Japan), which was stereotaxically introduced using the coordinates according to an atlas of the rat brain^52^. The anteroposterior, mediolateral, and dorsoventral coordinates (millimetres) from the bregma and dura were 1.5/3/3 (site 1), 1.5/3/3.5 (site 2), 0.5/3/3 (site 3), 0.5/3/3.5 (site 4), 1.5/−3/3 (site 5), 1.5/−3/3.5 (site 6), 0.5/−3/3 (site 7), and 0.5/−3/3.5 (site 8). Injection was performed at a constant flow rate of 0.1 μl/min using a micro-infusion pump. For the slice patch clamp experiment, the rats underwent the surgery when 21–40 days old. The animals were anaesthetized using ketamine/xylazine (50 and 10 mg/kg, respectively, intramuscularly) and placed in a stereotaxic frame. The scalp was incised and a 1–2-mm craniotomy was made over each injection coordinate point. The AAV2 virus expressing Cre under the cytomegalovirus promoter (4.5 × 10^11^ vector genomes/mL) was used as previously described^53^. The virus (1.5 μl) was pressure injected at each injection point in the hippocampus. The scalp was closed using a fast-acting cyanoacrylate adhesive (Aron alpha A; Sankyo, Tokyo, Japan).

### Primary culture of cortical neurons

Female LE rats were mated with male ROSA26/CAG-floxed STOP-ChRFR(C167A)-Venus BAC rats, and cortical neurons were isolated from the embryos at day 16. Cortical tissue was dissociated using a 2.5% Trypsin and 0.5% DNase I mixture and grown in culture medium (Sumitomo Bakelite, Tokyo, Japan) under a 5% CO_2_ atmosphere at 37 °C. The expression plasmid mCherry-NCre was transiently transfected in cortical neurons using calcium phosphate transfection at 6–7 days *in vitro* (DIV). Electrophysiological recordings were subsequently conducted at 21–25 DIV in neurons identified to express mCherry fluorescence under a conventional epifluorescence system.

To evaluate the recombination efficiency, 25 fields (218.9 μm^2^) of interest showing mCherry positive nuclei from wells (Nunc, cat. no. 176740) in each group were selected. Double blind counting was applied to detect cells positive for both Venus enhanced by Alexa-488 and mCherry enhanced by Alexa-546.

### Electrophysiology

All experiments were conducted at room temperature (23 ± 2 °C). Photocurrents were recorded as previously described^3^ using an EPC-8 amplifier (HEKA Electronic, Lambrecht, Germany) under a whole-cell patch clamp configuration. The data were filtered at 1 kHz, sampled at 100 kHz (Digdata1440 A/D, Molecular Devices Co., Sunnyvale, CA) and stored in a computer (pClamp10.3, Molecular Devices). The internal pipette solution for the whole-cell voltage-clamp and current-clamp recordings from primary cultured cortical neurons contained (in mM) 4 KOH, 2 MgCl_2_, 20 KCl, 115 K-gluconate, 0.2 EGTA, 10 HEPES, 5 Mg-ATP, 0.3 Na_2_GTP, 20 phosphocreatine and 50 U/ml creatine phosphokinase adjusted to pH 7.3 with KOH. The extracellular ACSF solution contained (in mM) 125 NaCl, 2.5 KCl, 25 NaHCO_3_, 1.25 NaH_2_PO_4_, 2 CaCl_2_, 1 MgCl_2_, 11 glucose, bubbled with mixed gas containing 95% O_2_ and 5% CO_2_. In all cortical neuron experiments, ACSF contained 3 mM kynurenic acid (Sigma, St. Louis, USA) and 100 μM picrotoxin (Nacalai, Kyoto, Japan) to block all synaptic inputs. Under whole-cell mode the resting potential was directly measured under current clamp and those neurons which had resting potentials of less than −50 mV served for the experiments (Supplementary Table S1). To investigate the excitability of a neuron the current ramp injection of 0.4–2 nA/s was made twice with an interval of 6 s through the patch electrode to depolarize the membrane potential above the threshold of generating action potentials.

### Optics

Irradiation was performed using a SpectraX light engine (Lumencor Inc., Beaverton, Oregon, USA) controlled by computer software (pCLAMP10.3, Molecular Devices) at wavelengths (nm, >90% of the maximum, maximal irradiance): 438 ± 24 (max. 3.0 mWmm^−2^, blue) and 643 ± 20 (max. 6.8 mWmm^−2^, red). The power density (irradiance) of the light was directly measured under microscopy by a visible light-sensing thermopile (MIR-101Q, SSC Co., Ltd., Kuwana City, Japan). To excite the primary culture neurons, blue irradiation was attenuated using ND filters at 50%, 25% and 5%. Closing kinetics of ChRFR(C167A) was evaluated using red light at the maximal irradiance (Supplementary Fig. S7). It followed a single exponential function with an OFF time constant (τ_OFF_) of 1080 ± 56 ms with a negligible component of the steady state photocurrent (*I*_*max*_ = −251 ± 46 nA, *C* = −17 ± 5 nA, n = 11). Therefore, the light-induced depolarization was almost completely attenuated by irradiating red light at the maximal irradiance for 8 s; the photocurrent was expected to be attenuated by 93.4% at the end of red light irradiation.

### Generation of transgenic Phox2b_tTA-2A-NLS-Cre BAC rats

The *Phox2b*_tTA-2A-NLS-Cre BAC (referred to as *Phox2b*_tTA-2A-Cre) transgenic construct was generated after integrating tandem cassettes of genes encoding a tetracycline transactivator (pTet-Off-Advanced, Clontech, Mountain View, CA), 2 A peptide, and Cre recombinase with a nuclear localization signal into clone 95M11 derived from the CHORI RP-24 C57BL/6 J (B6) mouse genomic library using the Red/ET recombination system (Gene Bridges GmbH, Heidelberg, Germany)^54^. A previous study showed that the 95M11 BAC clone contained the necessary regulatory regions required for the proper expression of the *Phox2b* gene in mice^55^. *Phox2b*_tTA-2A-Cre transgenic rats were generated by the pronuclear injection of Wistar rat embryos (Charles River Laboratory Japan Inc., Japan). Transgenic founders carrying the transgenic construct were assessed using *Southern* blotting. No obvious gross phenotypic differences were apparent between the transgene-positive and transgene-negative littermates. Several transgenic founder rats were bred with Wistar rats. Subsequent analyses were performed using one line (line 3_19). *Phox2b*_tTA-2A-Cre rats were mated with ROSA26/CAG-floxed STOP-ChRFR(C167A)-Venus BAC rats, and the newborn rats were used in subsequent experiments.

All animal experiments were approved by the Tohoku University Committee for Animal Experiments (Approval No. 2014LsA-023) and the Animal Research Committee of Showa University (56010). Experiments were performed in accordance with the Guidelines for Animal Experiments and Related Activities of Tohoku University and the guiding principles of the Physiological Society of Japan and the National Institutes of Health (NIH), USA. The number of animals in the present study was kept to a minimum. To minimize suffering, the rats were deeply anaesthetized with isoflurane in a glass bottle until nociceptive reflexes induced by tail pinch were completely abolished. Animals were provided access to food and water ad libitum and maintained under a 12-hour light-dark cycle.

### *Ex vivo* electrophysiology

Experiments were performed with brainstem-spinal cord preparations from newborn pups (0 to 3 days old) obtained after crossing Phox2b_tTA-2A-Cre RecBAC rats with ROSA26/CAG-floxed STOP-ChRFR(C167A)-Venus BAC rats. The newborn rats were deeply anaesthetized with isoflurane, and the brainstem and spinal cord were isolated as previously described^56–58^. In most experiments, the preparations were cut transversely at a level just rostral to the anterior inferior cerebellar artery, corresponding to the level between the roots of the sixth cranial nerve and the lower border of the trapezoid body. The preparations were continuously superfused with artificial cerebrospinal fluid (ACSF^56^) (composition [in mM]: 124 NaCl, 5 KCl, 1.2 KH_2_PO_4_, 2.4 CaCl_2_, 1.3 MgCl_2_, 26 NaHCO_3_, 30 glucose, equilibrated with 95% O_2_ and 5% CO_2_, pH 7.4) at a rate of 2.5–3 ml/min in a 2-ml chamber and maintained at a temperature of 25–26 °C. The inspiratory activity corresponding to phrenic nerve activity was monitored from the fourth cervical ventral root (C4). The rostral ventrolateral medulla corresponding to the pFRG was photostimulated by blue LED (460–470 nm, 0.1–0.2 mWmm^−2^) via an optic fibre with a 0.25-mm outer diameter for up to 35 s (50 ms duration/150 ms interval). The membrane potential was −44.8 ± 3.9 mV, and input resistance was 683 ± 205 MΩ (n = 6). The recorded neurons located in the rostral pFRG were labelled with Lucifer Yellow and identified as Phox2b positive. All data analyses were performed using LabChart 7 Pro software (ADInstruments, Castle Hill, Australia).

### Immunohistochemistry

Adult rat brains were resected three weeks after AAV2-Cre injection and promptly sectioned at 20 μm using a VT 1000S vibratome (Leica). Tissue slices were fixed with 4% paraformaldehyde, reacted with anti-Cre (1:500, MAB3120, Millipore) and anti-GFP (1:1000, 04404–84, Nacalai) antibodies, followed by secondary antibodies conjugated with Alexa Fluor 546 (1:200, Z25004, Molecular Probes, Eugene, OR, USA) and Alexa Fluor 488 (1:200, A11006, Molecular Probes, Eugene, OR, USA), respectively. Immunoreactivity was assessed under conventional fluorescence microscopy (Axiovert200, Carl Zeiss) or confocal microscopy (LSM510META, Carl Zeiss or FV1200, Olympus). The brainstem-spinal cords of rat neonates were also subjected to immunostaining after electrophysiological study. The preparations were fixed in 4% paraformaldehyde/0.1 M phosphate-buffered saline (PBS, pH 7.4) at 4 °C for 3 hrs. and immersed in 18% sucrose/PBS, embedded in Tissue-Tek optimal cutting temperature (OCT) compound (Sakura Finetek, Torrance, CA), frozen on dry ice, and subsequently cut into 18-μm-thick sections, followed by immunofluorescence imaging. Anti-Phox2b (1:2000 dilution^33^) and anti-Lucifer Yellow (Thermo Fisher Scientific/Invitrogen, Waltham, MA) were used for the primary antibodies, and Cy3-conjugated anti-guinea pig (Jackson ImmunoResearch, Baltimore Pike, West Grove, PA) and Alexa Fluor 488 anti-rabbit (Molecular Probes/Thermo Fisher Scientific, Eugene, OR) were used for the secondary antibodies. Images of the immunofluorescence samples were obtained using the 4× or 20× objectives of a BX51 fluorescence microscope (Olympus Optical). In each figure, the top is the dorsal side. The experiments were performed several times using different neonates, and the results were compatible between samples. Representative results are shown in the figures.

### Statistical analysis

All data in the text, figures and tables are expressed as mean ± SEM and were evaluated with the Mann-Whitney *U*-test for statistical significance unless otherwise noted. It was judged as statistically insignificant when P > 0.05.

## Electronic supplementary material

Dataset 1
