# Supplementary material for: Targeted expression of step-function opsins in transgenic rats for optogenetic studies
Source: Sci Rep. 2018 Apr 3;8:5435. doi: 10.1038/s41598-018-23810-8 (PMC5882906; doi:10.1038/s41598-018-23810-8)
Supplement: Supplementary file 1 — Dataset 1 [file 41598_2018_23810_MOESM1_ESM.zip › Supplemental data SFO rat-revise 2nd.pptx]

## Slide 1
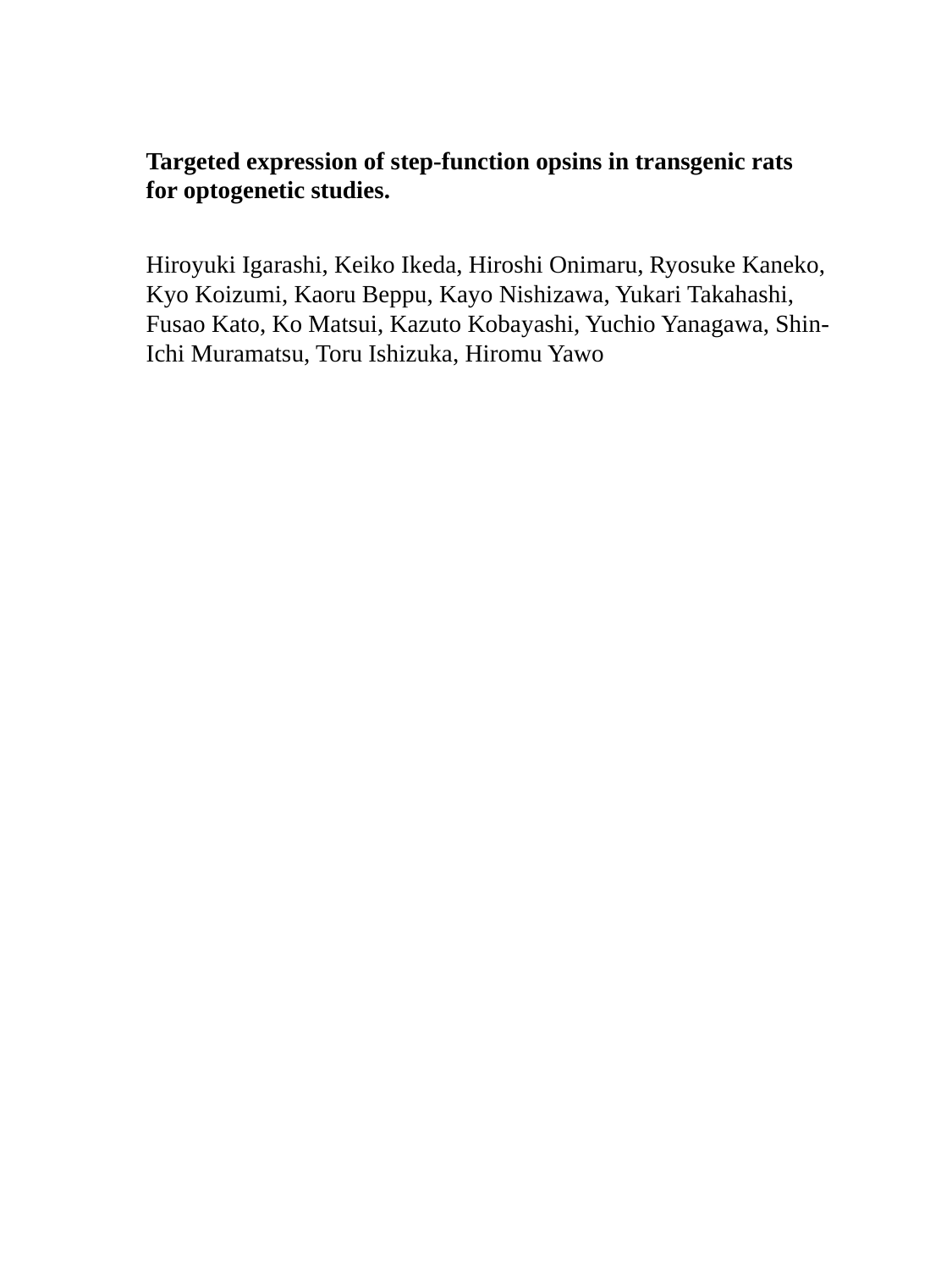

Targeted expression of step-function opsins in transgenic rats for optogenetic studies.
Hiroyuki Igarashi, Keiko Ikeda, Hiroshi Onimaru, Ryosuke Kaneko, Kyo Koizumi, Kaoru Beppu, Kayo Nishizawa, Yukari Takahashi, Fusao Kato, Ko Matsui, Kazuto Kobayashi, Yuchio Yanagawa, Shin-Ichi Muramatsu, Toru Ishizuka, Hiromu Yawo

## Slide 2
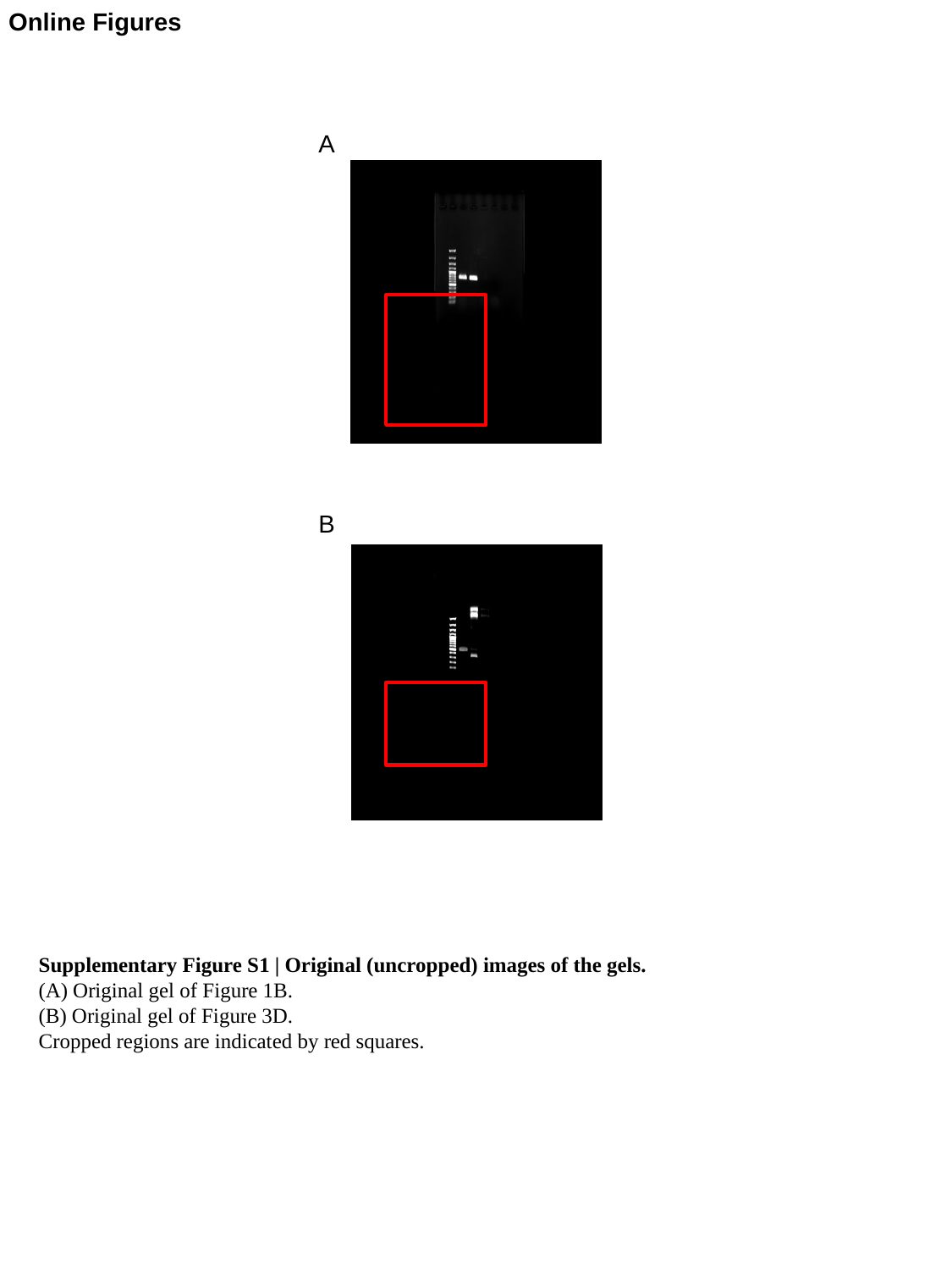

Online Figures
A
B
Supplementary Figure S1 | Original (uncropped) images of the gels.
(A) Original gel of Figure 1B.
(B) Original gel of Figure 3D.
Cropped regions are indicated by red squares.

## Slide 3
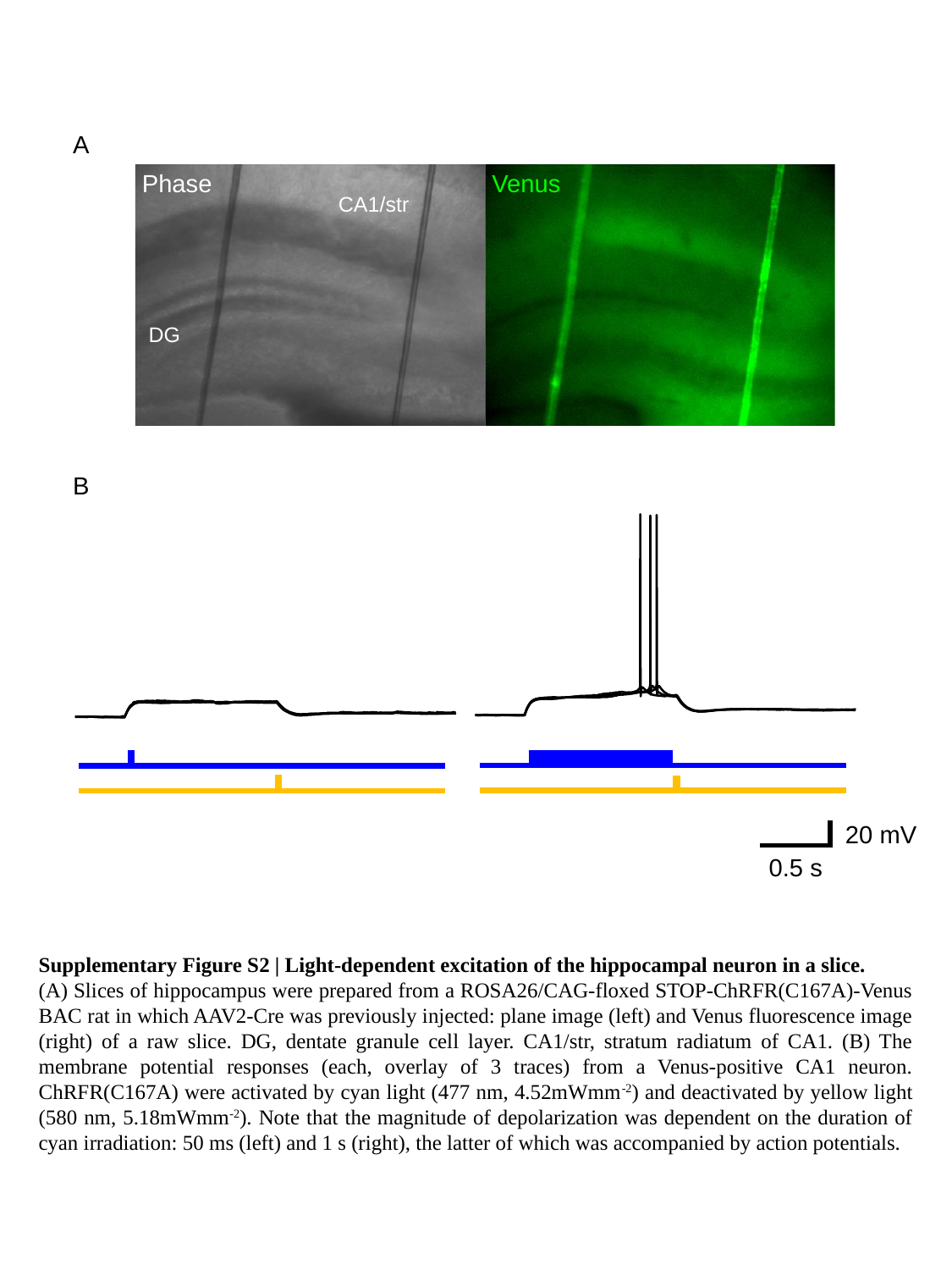

A
Phase
Venus
CA1/str
DG
B
### Chart
| Category | 1 10 Im00 Trace #1 (pA) | 1 10 Im00 Trace #2 (pA) | 1 10 Im00 Trace #3 (pA) |
|---|---|---|---|
### Chart
| Category | 1 10 Im00 Trace #1 (pA) | 1 10 Im00 Trace #2 (pA) | 1 10 Im00 Trace #3 (pA) |
|---|---|---|---|20 mV
0.5 s
Supplementary Figure S2 | Light-dependent excitation of the hippocampal neuron in a slice.
(A) Slices of hippocampus were prepared from a ROSA26/CAG-floxed STOP-ChRFR(C167A)-Venus BAC rat in which AAV2-Cre was previously injected: plane image (left) and Venus fluorescence image (right) of a raw slice. DG, dentate granule cell layer. CA1/str, stratum radiatum of CA1. (B) The membrane potential responses (each, overlay of 3 traces) from a Venus-positive CA1 neuron. ChRFR(C167A) were activated by cyan light (477 nm, 4.52mWmm-2) and deactivated by yellow light (580 nm, 5.18mWmm-2). Note that the magnitude of depolarization was dependent on the duration of cyan irradiation: 50 ms (left) and 1 s (right), the latter of which was accompanied by action potentials.

## Slide 4
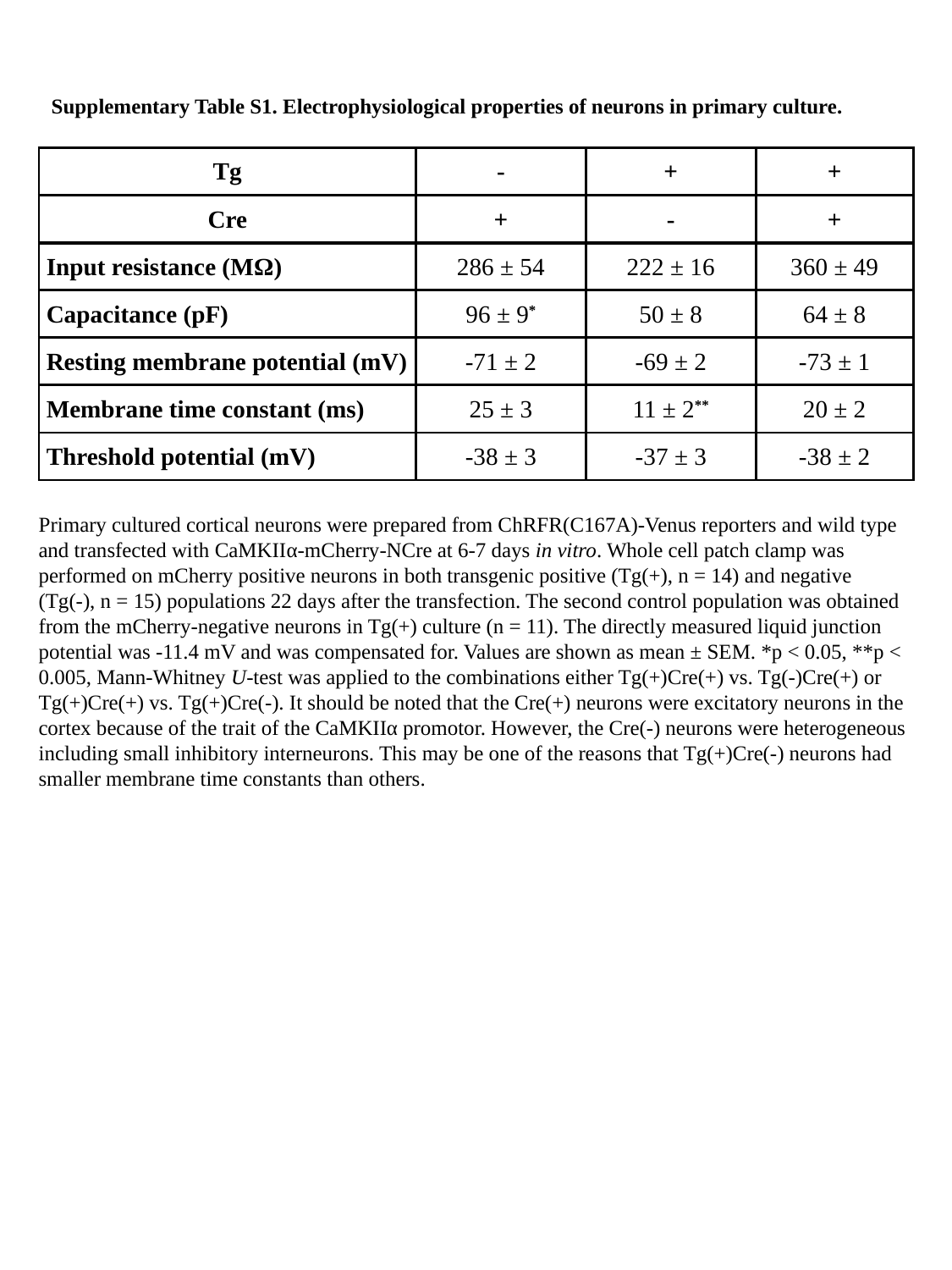

Supplementary Table S1. Electrophysiological properties of neurons in primary culture.
| Tg | - | + | + |
| --- | --- | --- | --- |
| Cre | + | - | + |
| Input resistance (MΩ) | 286 ± 54 | 222 ± 16 | 360 ± 49 |
| Capacitance (pF) | 96 ± 9\* | 50 ± 8 | 64 ± 8 |
| Resting membrane potential (mV) | -71 ± 2 | -69 ± 2 | -73 ± 1 |
| Membrane time constant (ms) | 25 ± 3 | 11 ± 2\*\* | 20 ± 2 |
| Threshold potential (mV) | -38 ± 3 | -37 ± 3 | -38 ± 2 |
Primary cultured cortical neurons were prepared from ChRFR(C167A)-Venus reporters and wild type and transfected with CaMKIIα-mCherry-NCre at 6-7 days in vitro. Whole cell patch clamp was performed on mCherry positive neurons in both transgenic positive (Tg(+), n = 14) and negative (Tg(-), n = 15) populations 22 days after the transfection. The second control population was obtained from the mCherry-negative neurons in Tg(+) culture (n = 11). The directly measured liquid junction potential was -11.4 mV and was compensated for. Values are shown as mean ± SEM. *p < 0.05, **p < 0.005, Mann-Whitney U-test was applied to the combinations either Tg(+)Cre(+) vs. Tg(-)Cre(+) or Tg(+)Cre(+) vs. Tg(+)Cre(-). It should be noted that the Cre(+) neurons were excitatory neurons in the cortex because of the trait of the CaMKIIα promotor. However, the Cre(-) neurons were heterogeneous including small inhibitory interneurons. This may be one of the reasons that Tg(+)Cre(-) neurons had smaller membrane time constants than others.

## Slide 5
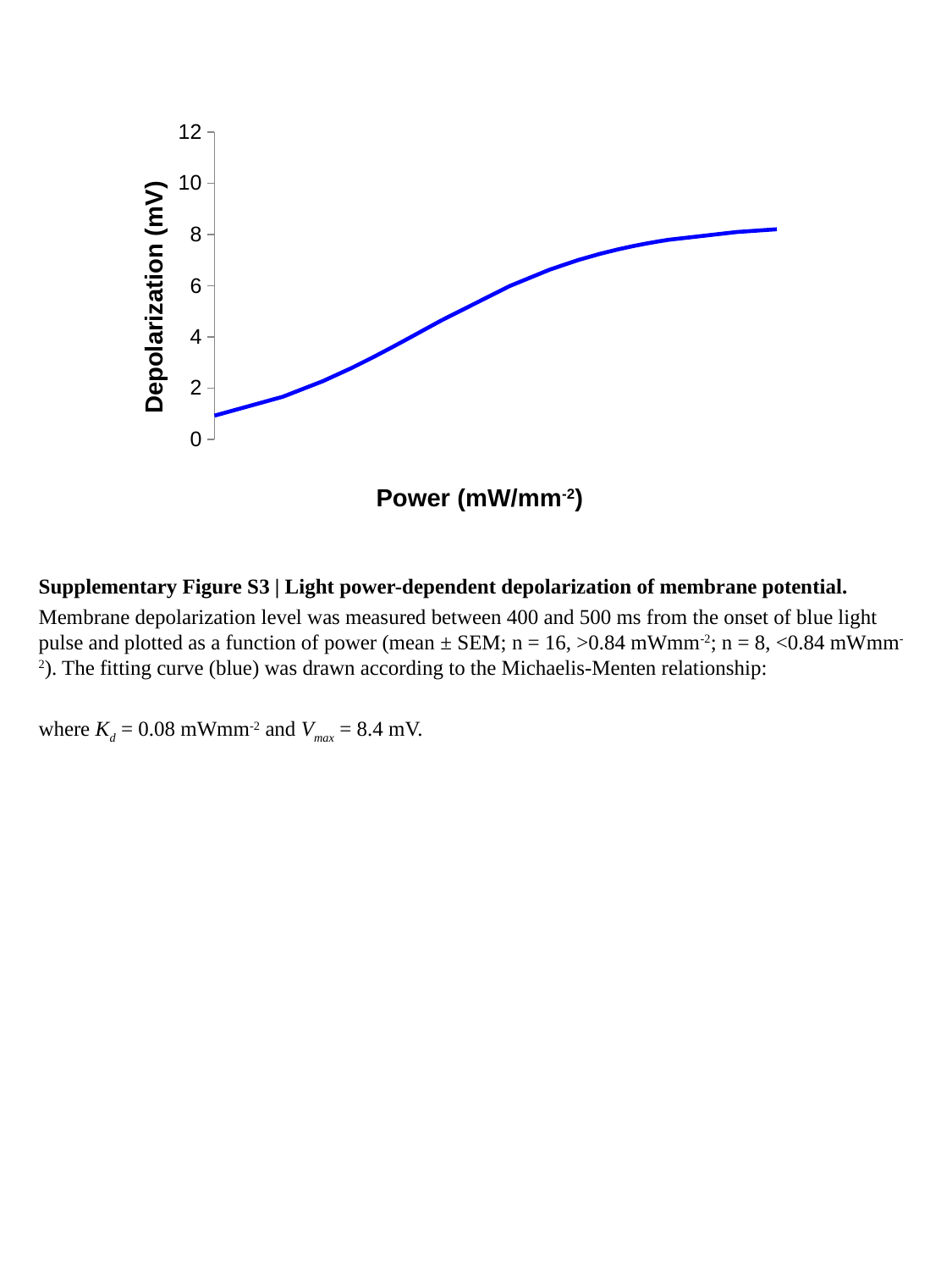

### Chart
| Category | | |
|---|---|---|Depolarization (mV)
Power (mW/mm-2)

## Slide 6
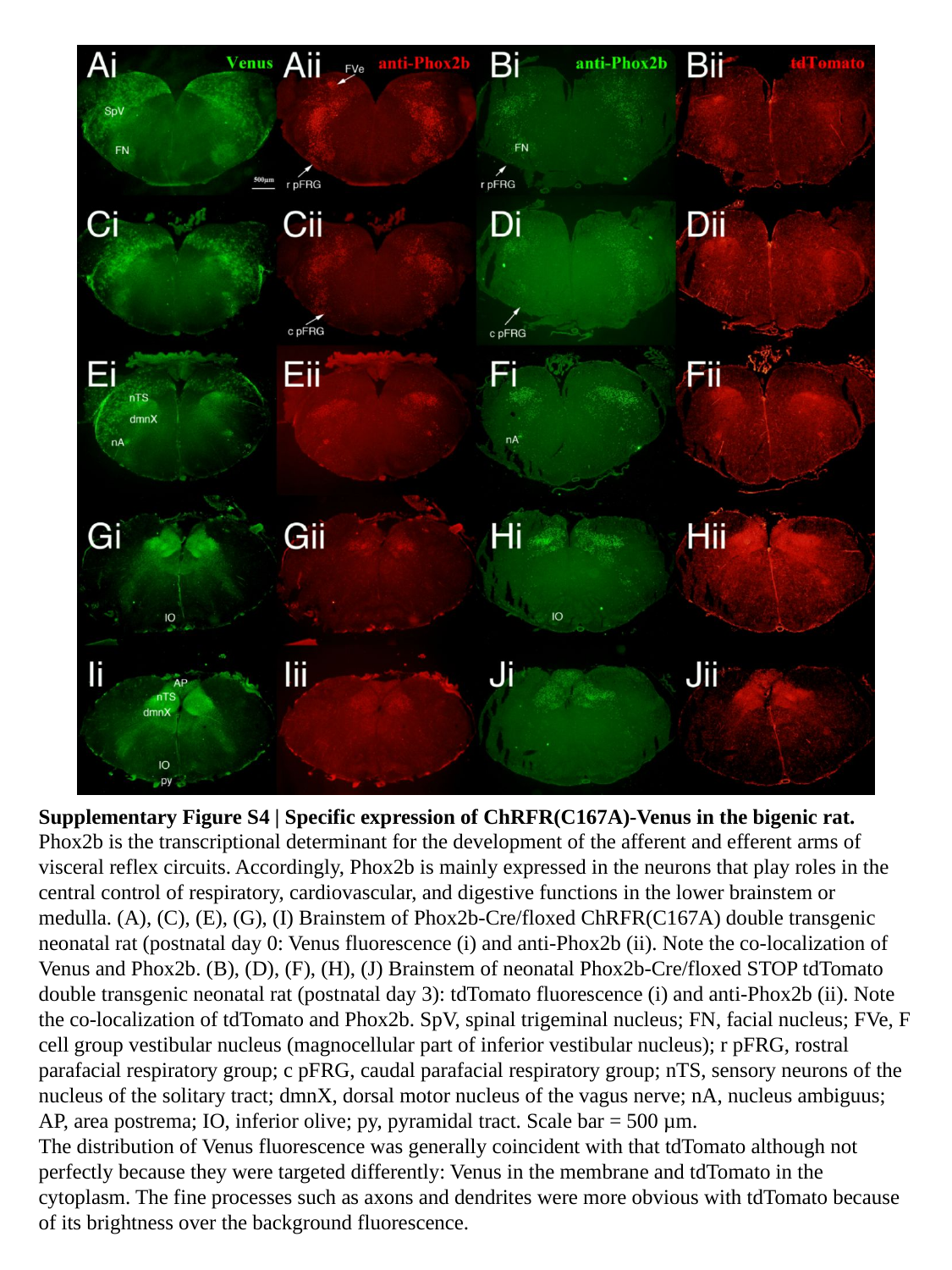

Supplementary Figure S4 | Specific expression of ChRFR(C167A)-Venus in the bigenic rat.
Phox2b is the transcriptional determinant for the development of the afferent and efferent arms of visceral reflex circuits. Accordingly, Phox2b is mainly expressed in the neurons that play roles in the central control of respiratory, cardiovascular, and digestive functions in the lower brainstem or medulla. (A), (C), (E), (G), (I) Brainstem of Phox2b-Cre/floxed ChRFR(C167A) double transgenic neonatal rat (postnatal day 0: Venus fluorescence (i) and anti-Phox2b (ii). Note the co-localization of Venus and Phox2b. (B), (D), (F), (H), (J) Brainstem of neonatal Phox2b-Cre/floxed STOP tdTomato double transgenic neonatal rat (postnatal day 3): tdTomato fluorescence (i) and anti-Phox2b (ii). Note the co-localization of tdTomato and Phox2b. SpV, spinal trigeminal nucleus; FN, facial nucleus; FVe, F cell group vestibular nucleus (magnocellular part of inferior vestibular nucleus); r pFRG, rostral parafacial respiratory group; c pFRG, caudal parafacial respiratory group; nTS, sensory neurons of the nucleus of the solitary tract; dmnX, dorsal motor nucleus of the vagus nerve; nA, nucleus ambiguus; AP, area postrema; IO, inferior olive; py, pyramidal tract. Scale bar = 500 µm.
The distribution of Venus fluorescence was generally coincident with that tdTomato although not perfectly because they were targeted differently: Venus in the membrane and tdTomato in the cytoplasm. The fine processes such as axons and dendrites were more obvious with tdTomato because of its brightness over the background fluorescence.

## Slide 7
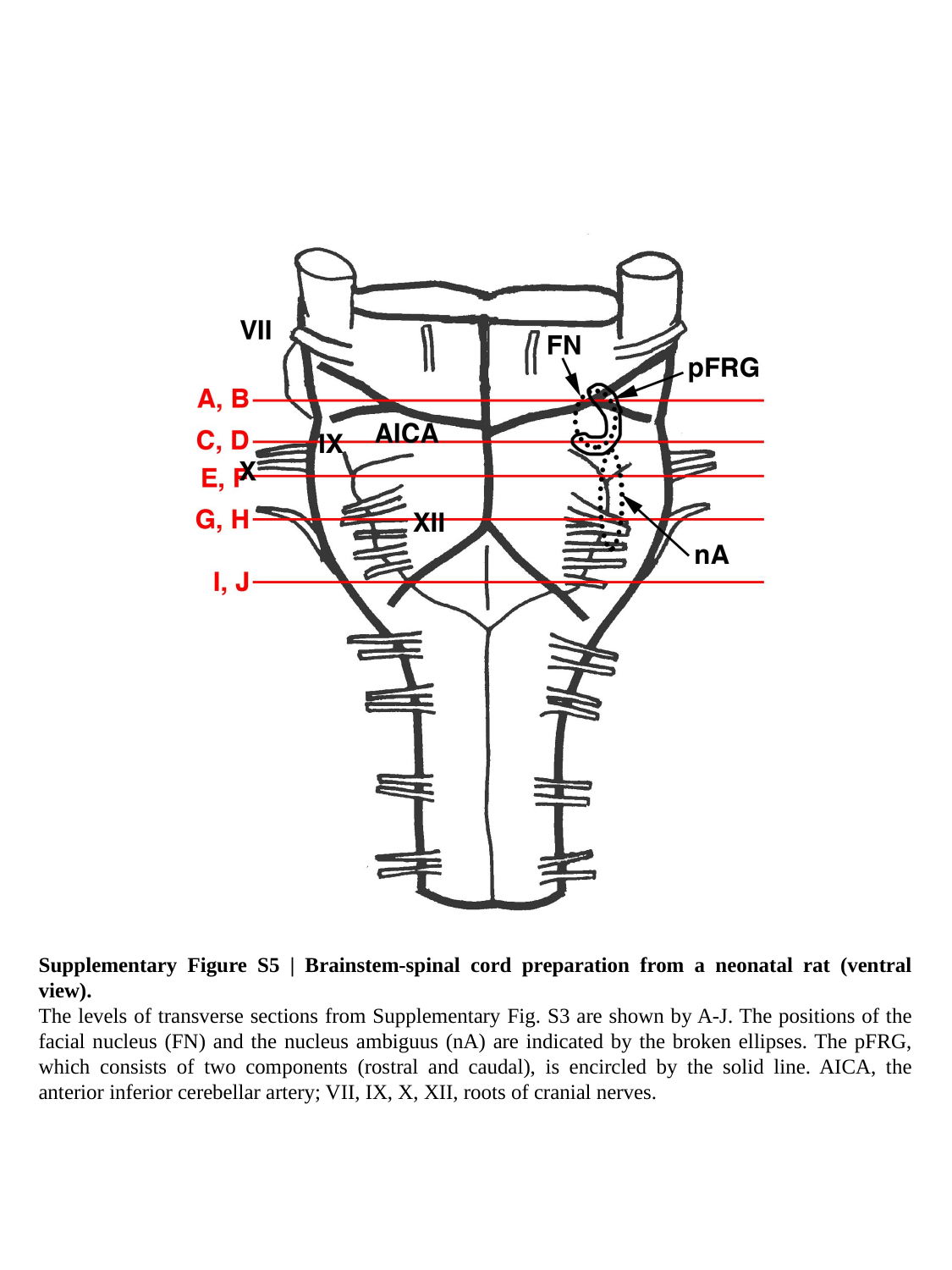

Supplementary Figure S5 | Brainstem-spinal cord preparation from a neonatal rat (ventral view).
The levels of transverse sections from Supplementary Fig. S3 are shown by A-J. The positions of the facial nucleus (FN) and the nucleus ambiguus (nA) are indicated by the broken ellipses. The pFRG, which consists of two components (rostral and caudal), is encircled by the solid line. AICA, the anterior inferior cerebellar artery; VII, IX, X, XII, roots of cranial nerves.

## Slide 8
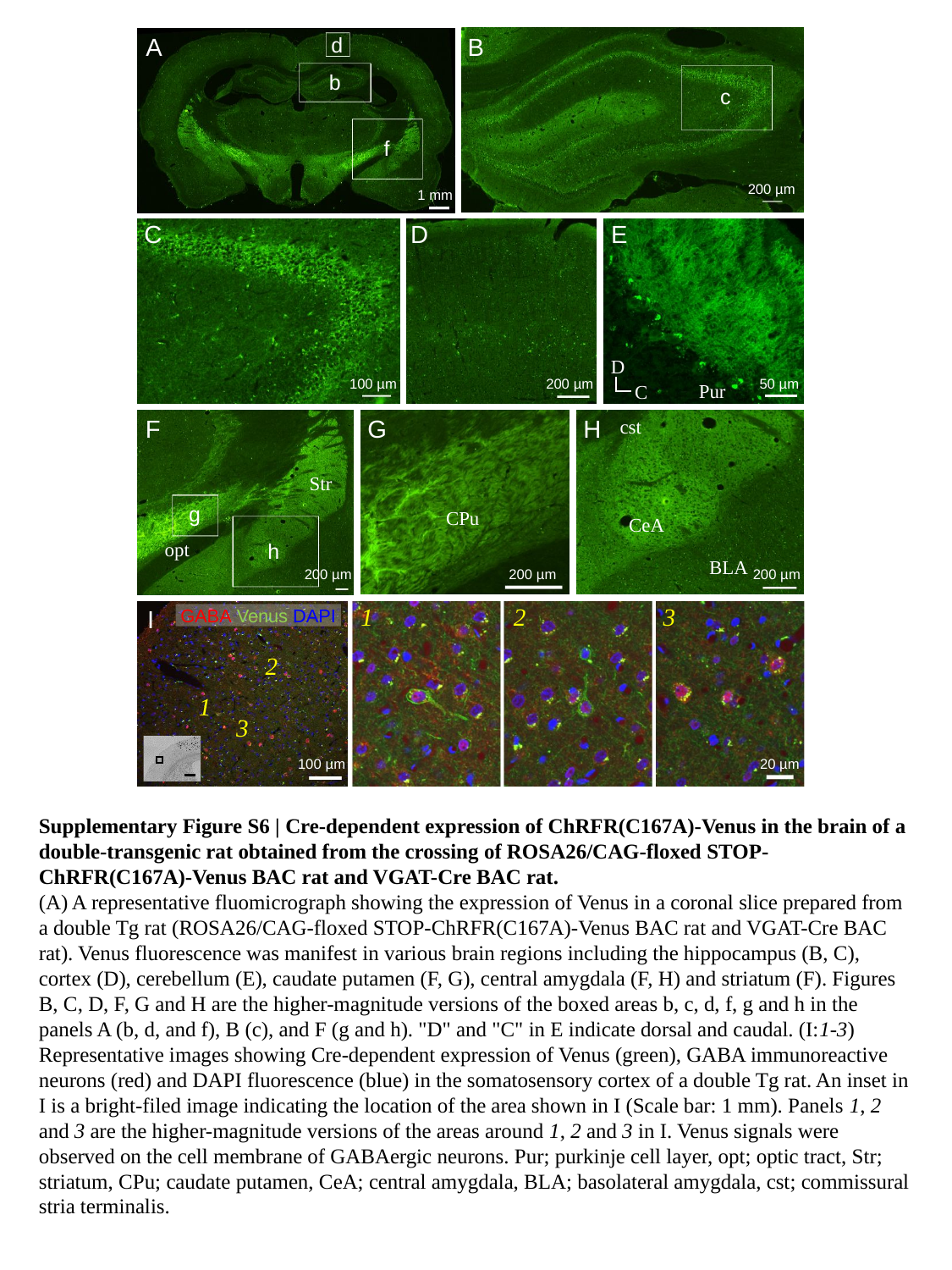

A
B
d
b
c
f
200 µm
1 mm
C
D
E
D
C
100 µm
200 µm
50 µm
Pur
F
G
H
cst
Str
g
CPu
CeA
opt
h
BLA
200 µm
200 µm
200 µm
1
2
3
I
GABA Venus DAPI
2
1
3
100 µm
20 µm
Supplementary Figure S6 | Cre-dependent expression of ChRFR(C167A)-Venus in the brain of a double-transgenic rat obtained from the crossing of ROSA26/CAG-floxed STOP-ChRFR(C167A)-Venus BAC rat and VGAT-Cre BAC rat.
(A) A representative fluomicrograph showing the expression of Venus in a coronal slice prepared from a double Tg rat (ROSA26/CAG-floxed STOP-ChRFR(C167A)-Venus BAC rat and VGAT-Cre BAC rat). Venus fluorescence was manifest in various brain regions including the hippocampus (B, C), cortex (D), cerebellum (E), caudate putamen (F, G), central amygdala (F, H) and striatum (F). Figures B, C, D, F, G and H are the higher-magnitude versions of the boxed areas b, c, d, f, g and h in the panels A (b, d, and f), B (c), and F (g and h). "D" and "C" in E indicate dorsal and caudal. (I:1-3) Representative images showing Cre-dependent expression of Venus (green), GABA immunoreactive neurons (red) and DAPI fluorescence (blue) in the somatosensory cortex of a double Tg rat. An inset in I is a bright-filed image indicating the location of the area shown in I (Scale bar: 1 mm). Panels 1, 2 and 3 are the higher-magnitude versions of the areas around 1, 2 and 3 in I. Venus signals were observed on the cell membrane of GABAergic neurons. Pur; purkinje cell layer, opt; optic tract, Str; striatum, CPu; caudate putamen, CeA; central amygdala, BLA; basolateral amygdala, cst; commissural stria terminalis.

## Slide 9
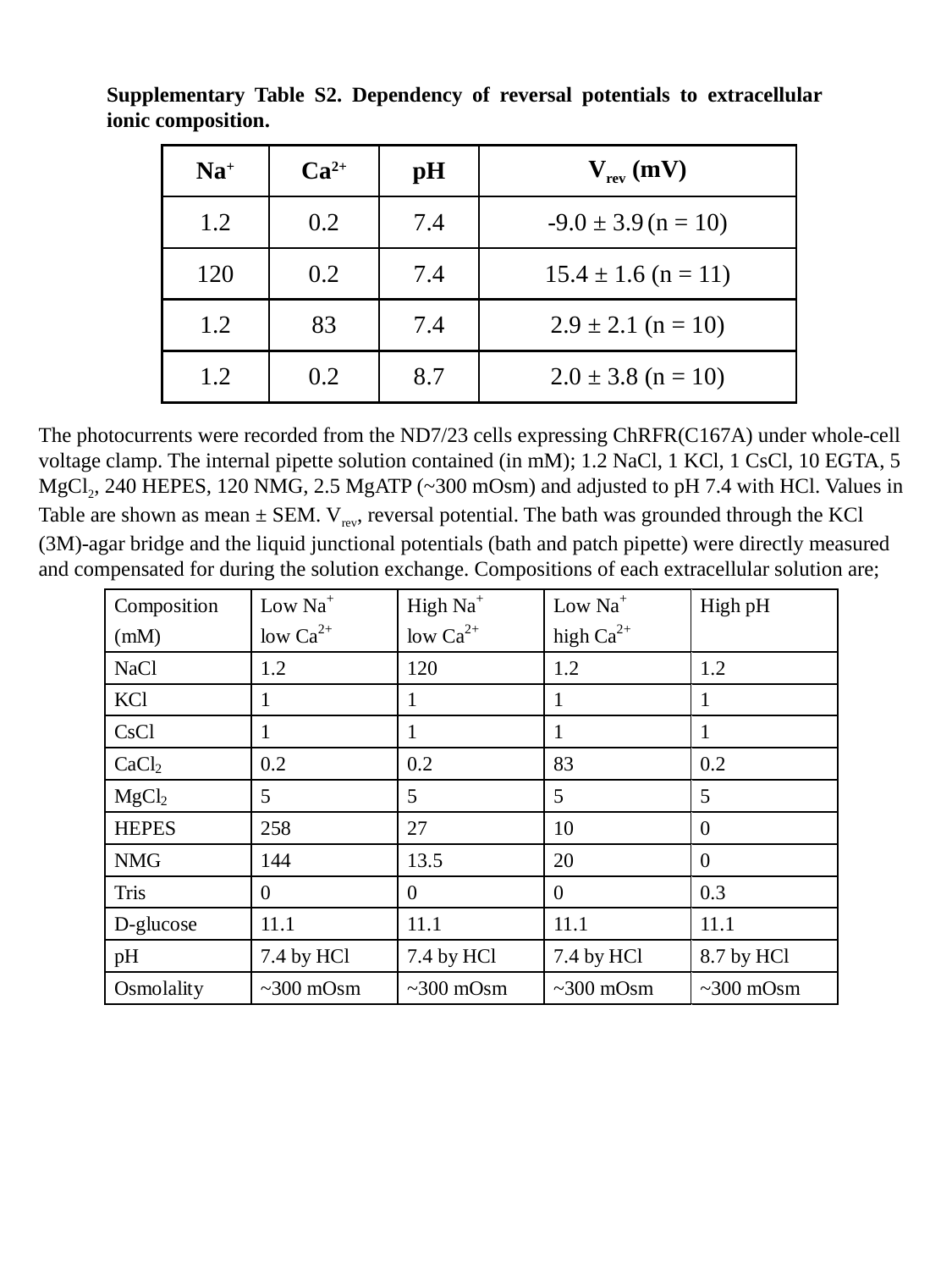

Supplementary Table S2. Dependency of reversal potentials to extracellular ionic composition.
| Na+ | Ca2+ | pH | Vrev (mV) |
| --- | --- | --- | --- |
| 1.2 | 0.2 | 7.4 | -9.0 ± 3.9 (n = 10) |
| 120 | 0.2 | 7.4 | 15.4 ± 1.6 (n = 11) |
| 1.2 | 83 | 7.4 | 2.9 ± 2.1 (n = 10) |
| 1.2 | 0.2 | 8.7 | 2.0 ± 3.8 (n = 10) |
The photocurrents were recorded from the ND7/23 cells expressing ChRFR(C167A) under whole-cell voltage clamp. The internal pipette solution contained (in mM); 1.2 NaCl, 1 KCl, 1 CsCl, 10 EGTA, 5 MgCl2, 240 HEPES, 120 NMG, 2.5 MgATP (~300 mOsm) and adjusted to pH 7.4 with HCl. Values in Table are shown as mean ± SEM. Vrev, reversal potential. The bath was grounded through the KCl (3M)-agar bridge and the liquid junctional potentials (bath and patch pipette) were directly measured and compensated for during the solution exchange. Compositions of each extracellular solution are;

## Slide 10
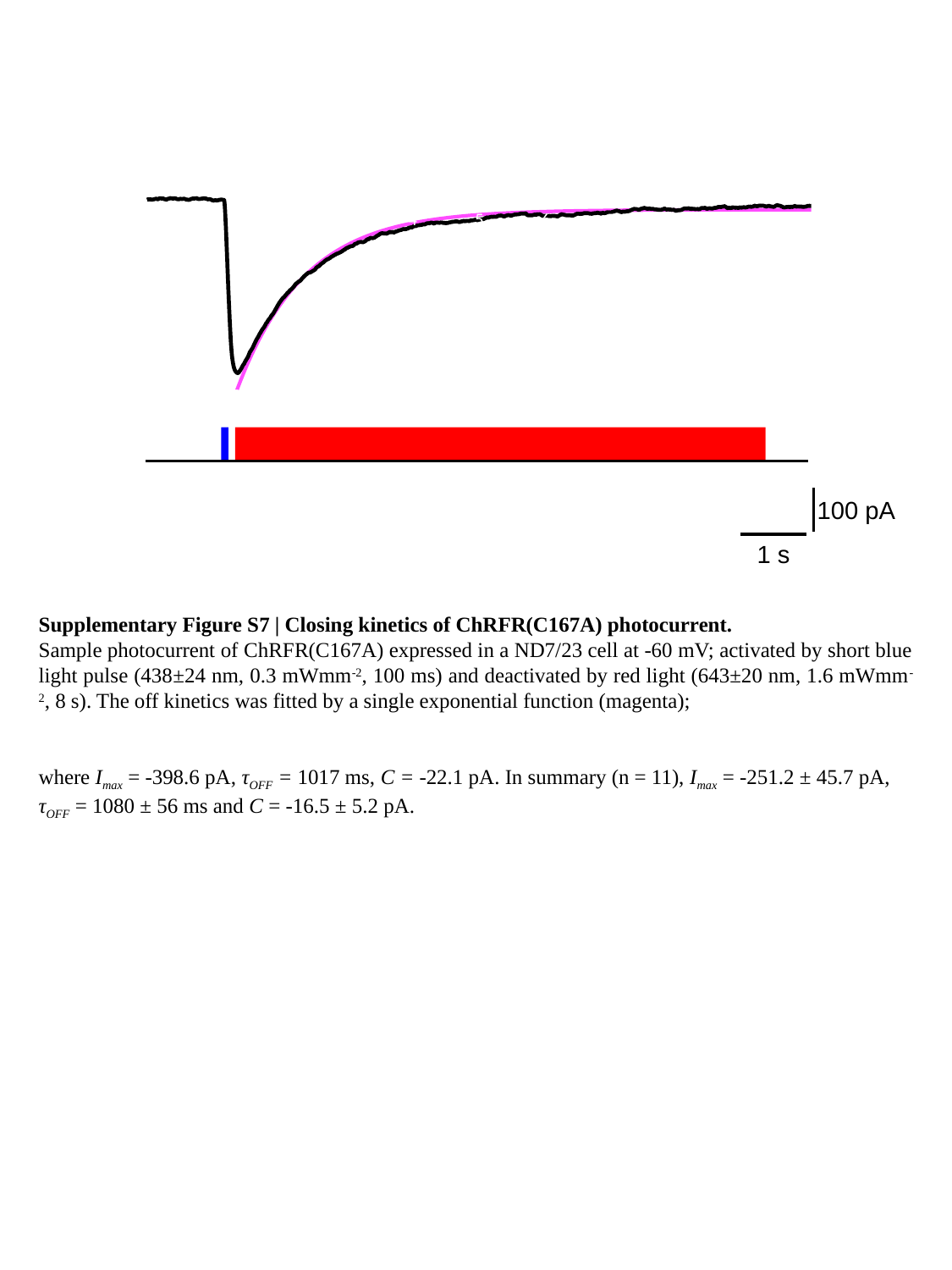

### Chart
| Category | | |
|---|---|---|
100 pA
1 s

## Slide 11
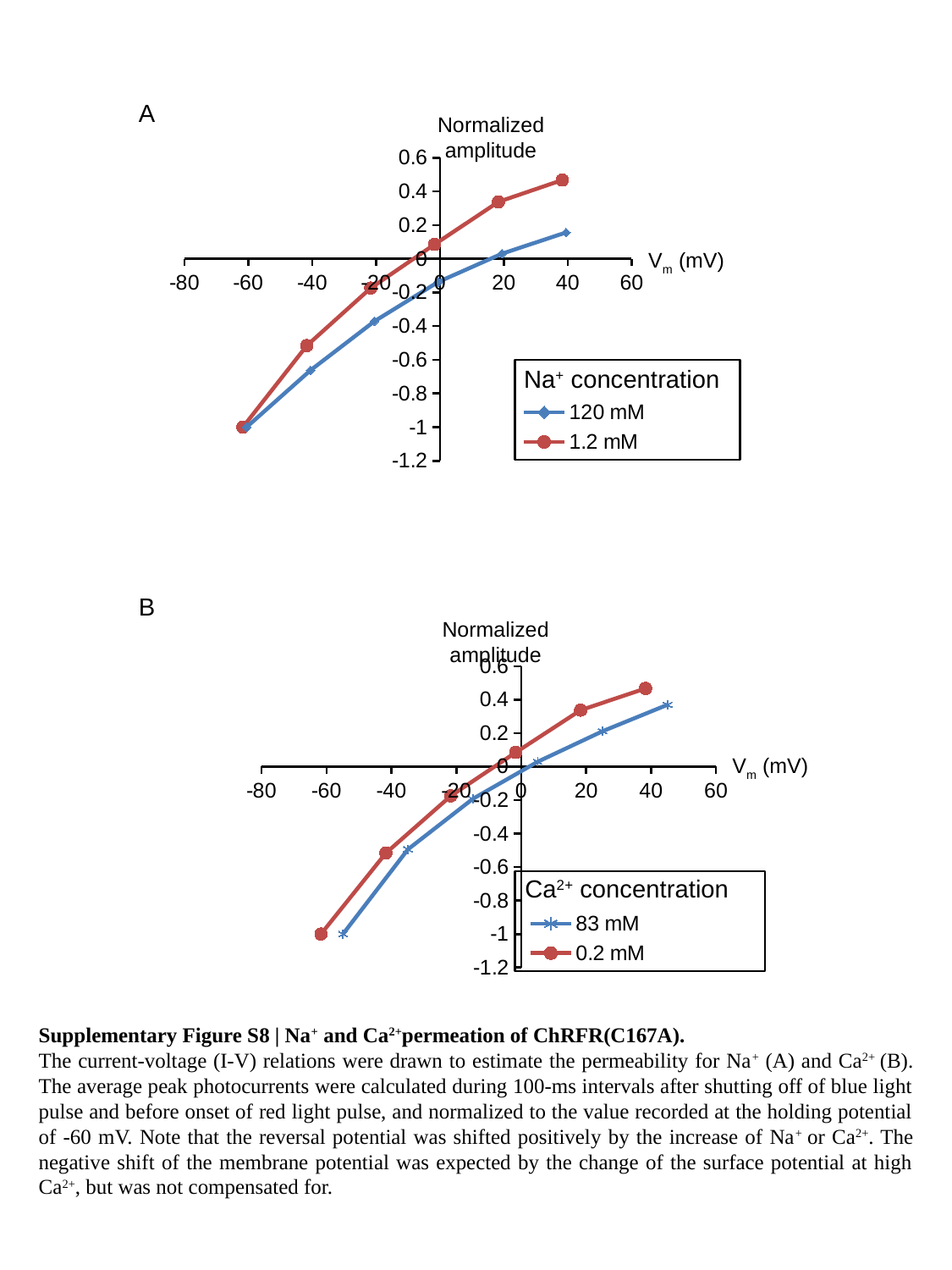

A
Normalized amplitude
### Chart
| Category | | |
|---|---|---|Vm (mV)
Na+ concentration
B
Normalized amplitude
### Chart
| Category | | |
|---|---|---|Vm (mV)
Ca2+ concentration
Supplementary Figure S8 | Na+ and Ca2+permeation of ChRFR(C167A).
The current-voltage (I-V) relations were drawn to estimate the permeability for Na+ (A) and Ca2+ (B). The average peak photocurrents were calculated during 100-ms intervals after shutting off of blue light pulse and before onset of red light pulse, and normalized to the value recorded at the holding potential of -60 mV. Note that the reversal potential was shifted positively by the increase of Na+ or Ca2+. The negative shift of the membrane potential was expected by the change of the surface potential at high Ca2+, but was not compensated for.

## Slide 12
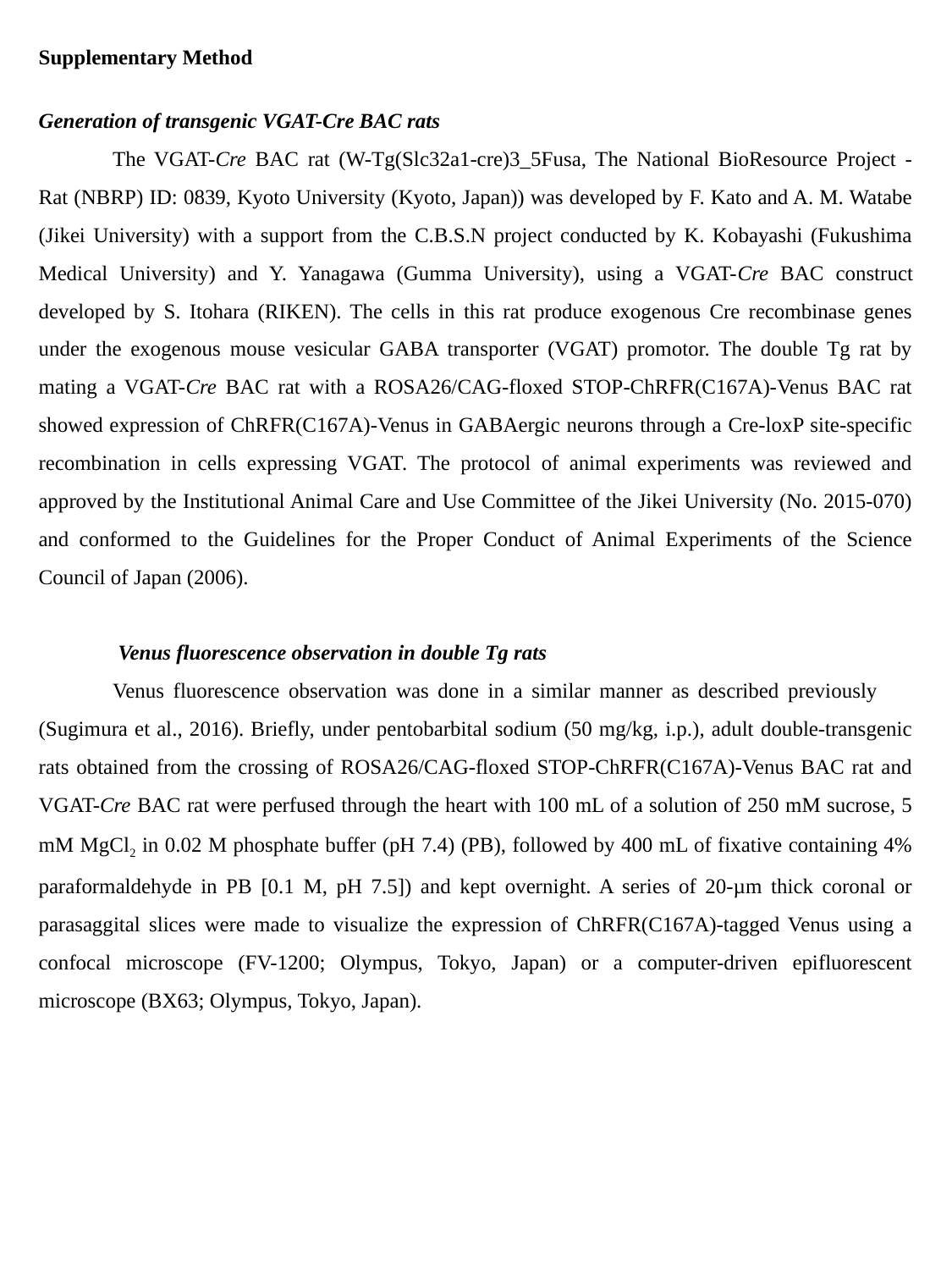

Supplementary Method
Generation of transgenic VGAT-Cre BAC rats
The VGAT-Cre BAC rat (W-Tg(Slc32a1-cre)3_5Fusa, The National BioResource Project - Rat (NBRP) ID: 0839, Kyoto University (Kyoto, Japan)) was developed by F. Kato and A. M. Watabe (Jikei University) with a support from the C.B.S.N project conducted by K. Kobayashi (Fukushima Medical University) and Y. Yanagawa (Gumma University), using a VGAT-Cre BAC construct developed by S. Itohara (RIKEN). The cells in this rat produce exogenous Cre recombinase genes under the exogenous mouse vesicular GABA transporter (VGAT) promotor. The double Tg rat by mating a VGAT-Cre BAC rat with a ROSA26/CAG-floxed STOP-ChRFR(C167A)-Venus BAC rat showed expression of ChRFR(C167A)-Venus in GABAergic neurons through a Cre-loxP site-specific recombination in cells expressing VGAT. The protocol of animal experiments was reviewed and approved by the Institutional Animal Care and Use Committee of the Jikei University (No. 2015-070) and conformed to the Guidelines for the Proper Conduct of Animal Experiments of the Science Council of Japan (2006).
 Venus fluorescence observation in double Tg rats
Venus fluorescence observation was done in a similar manner as described previously　(Sugimura et al., 2016). Briefly, under pentobarbital sodium (50 mg/kg, i.p.), adult double-transgenic rats obtained from the crossing of ROSA26/CAG-floxed STOP-ChRFR(C167A)-Venus BAC rat and VGAT-Cre BAC rat were perfused through the heart with 100 mL of a solution of 250 mM sucrose, 5 mM MgCl2 in 0.02 M phosphate buffer (pH 7.4) (PB), followed by 400 mL of fixative containing 4% paraformaldehyde in PB [0.1 M, pH 7.5]) and kept overnight. A series of 20-µm thick coronal or parasaggital slices were made to visualize the expression of ChRFR(C167A)-tagged Venus using a confocal microscope (FV-1200; Olympus, Tokyo, Japan) or a computer-driven epifluorescent microscope (BX63; Olympus, Tokyo, Japan).

## Slide 13
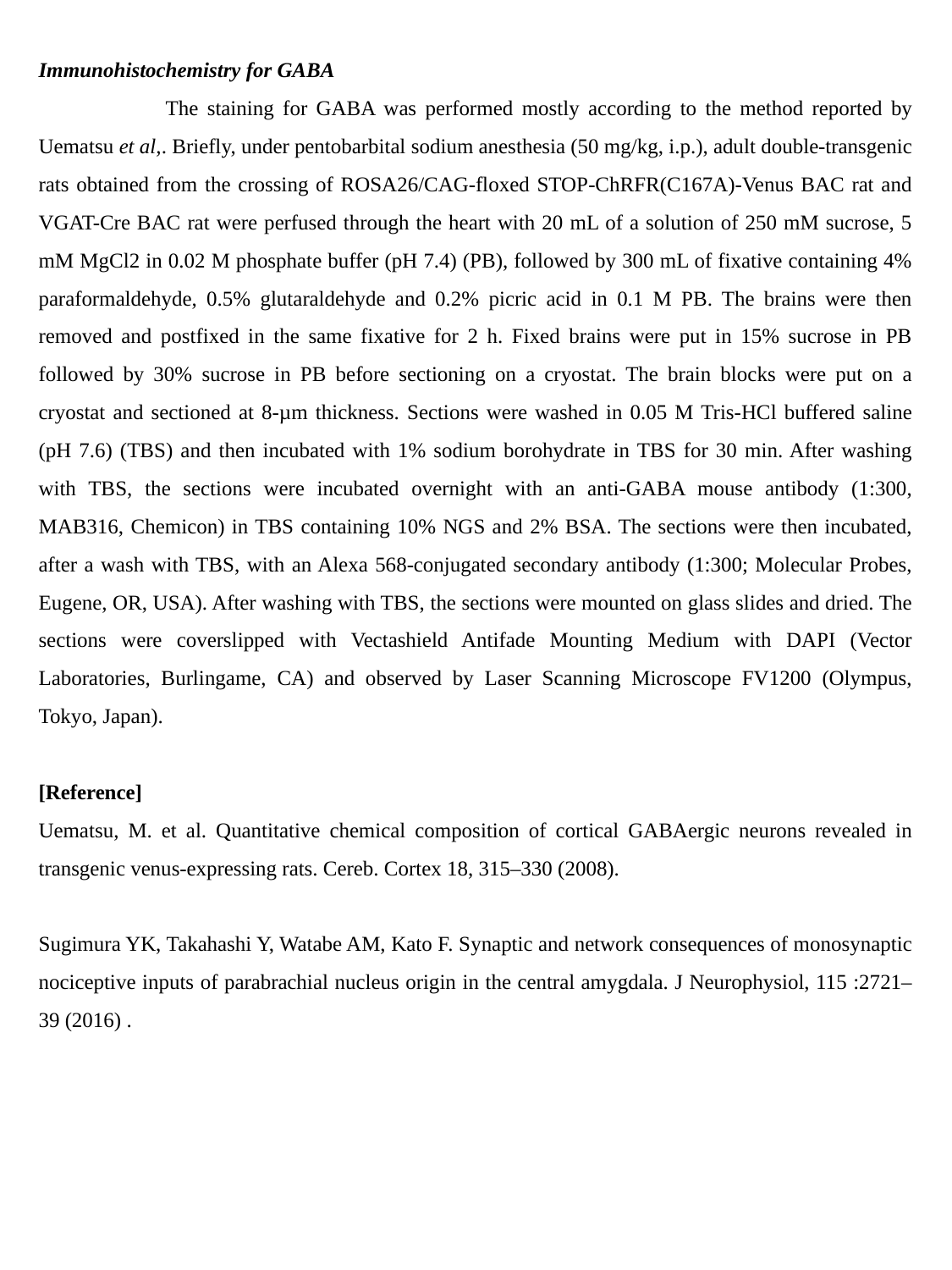

Immunohistochemistry for GABA
	The staining for GABA was performed mostly according to the method reported by Uematsu et al,. Briefly, under pentobarbital sodium anesthesia (50 mg/kg, i.p.), adult double-transgenic rats obtained from the crossing of ROSA26/CAG-floxed STOP-ChRFR(C167A)-Venus BAC rat and VGAT-Cre BAC rat were perfused through the heart with 20 mL of a solution of 250 mM sucrose, 5 mM MgCl2 in 0.02 M phosphate buffer (pH 7.4) (PB), followed by 300 mL of fixative containing 4% paraformaldehyde, 0.5% glutaraldehyde and 0.2% picric acid in 0.1 M PB. The brains were then removed and postfixed in the same fixative for 2 h. Fixed brains were put in 15% sucrose in PB followed by 30% sucrose in PB before sectioning on a cryostat. The brain blocks were put on a cryostat and sectioned at 8-µm thickness. Sections were washed in 0.05 M Tris-HCl buffered saline (pH 7.6) (TBS) and then incubated with 1% sodium borohydrate in TBS for 30 min. After washing with TBS, the sections were incubated overnight with an anti-GABA mouse antibody (1:300, MAB316, Chemicon) in TBS containing 10% NGS and 2% BSA. The sections were then incubated, after a wash with TBS, with an Alexa 568-conjugated secondary antibody (1:300; Molecular Probes, Eugene, OR, USA). After washing with TBS, the sections were mounted on glass slides and dried. The sections were coverslipped with Vectashield Antifade Mounting Medium with DAPI (Vector Laboratories, Burlingame, CA) and observed by Laser Scanning Microscope FV1200 (Olympus, Tokyo, Japan).
[Reference]
Uematsu, M. et al. Quantitative chemical composition of cortical GABAergic neurons revealed in transgenic venus-expressing rats. Cereb. Cortex 18, 315–330 (2008).
Sugimura YK, Takahashi Y, Watabe AM, Kato F. Synaptic and network consequences of monosynaptic nociceptive inputs of parabrachial nucleus origin in the central amygdala. J Neurophysiol, 115 :2721–39 (2016) .
